# *Raptor* determines β-cell identity and plasticity independent of hyperglycemia in mice

**DOI:** 10.1038/s41467-020-15935-0

**Published:** 2020-05-21

**Authors:** Qinglei Yin, Qicheng Ni, Yichen Wang, Hongli Zhang, Wenyi Li, Aifang Nie, Shu Wang, Yanyun Gu, Qidi Wang, Guang Ning

**Affiliations:** 1 Shanghai National Clinical Research Center for Endocrine and Metabolic Diseases, Key Laboratory for Endocrine and Metabolic Diseases of the National Health Commission of the PR China, Shanghai Institute of Endocrine and Metabolic Diseases, Ruijin Hospital, Shanghai Jiao Tong University School of Medicine, 200025 Shanghai, China; 2 Department of Endocrinology, Seventh People’s Hospital of Shanghai University of Traditional Chinese Medicine, 200137 Shanghai, China; 3 Sino-French Research Center for Life Sciences and Genomics, Ruijin Hospital Affiliated to Shanghai Jiao Tong University School of Medicine, 200025 Shanghai, China

**Keywords:** Cell signalling, Mechanisms of disease, Diabetes, Endocrine system and metabolic diseases

## Abstract

Compromised β-cell identity is emerging as an important contributor to β-cell failure in diabetes; however, the precise mechanism independent of hyperglycemia is under investigation. We have previously reported that mTORC1/*Rapto*r regulates functional maturation in β-cells. In the present study, we find that diabetic β-cell specific *Raptor*-deficient mice (βRapKO^GFP^) show reduced β-cell mass, loss of β-cell identity and acquisition of α-cell features; which are not reversible upon glucose normalization. Deletion of *Raptor* directly impairs β-cell identity, mitochondrial metabolic coupling and protein synthetic activity, leading to β-cell failure. Moreover, loss of *Raptor* activates α-cell transcription factor *MafB* (via modulating C/EBPβ isoform ratio) and several α-cell enriched genes i.e. *Etv1* and *Tspan12*, thus initiates β- to α-cell reprograming. The present findings highlight mTORC1 as a metabolic rheostat for stabilizing β-cell identity and repressing α-cell program at normoglycemic level, which might present therapeutic opportunities for treatment of diabetes.

## Abstract (web summary)

During the progression of type 2 diabetes, insulin-producing β-cells can lose their identity and become reprogrammed into other cell types. Here the authors show that murine diabetic β-cells require the protein Raptor for maintaining β-cell health and preventing them from turning into α-cells, independent of Raptor’s involvement in regulating blood sugar levels.

## Introduction

A central feature of type 2 diabetes (T2D) is progressive loss of functional β-cell mass, which ultimately results in β-cell failure and insulin insufficiency to meet metabolic demands^1^. The development of β-cell failure can be formulated into three stages: (1) healthy state, (2) impaired coupling of cellular metabolism with insulin secretion, and finally (3) loss of features as a mature, hormone-laden cell (dedifferentiation) with conversion to other endocrine cell types^2^. It was proposed that loss of β-cell identity might be one of the mechanisms of loss of functional β-cell mass in diabetes^3,4^. Deletion of several important transcription factors, i.e. *Foxo1* (ref. ^5^), *Pdx1* (ref. ^6^), *Nkx6.1* (ref. ^7^), *MafA*^8^, *Nkx2.2* (ref. ^9^), *Pax6* (ref. ^10^) perturbs β-cell identity by silencing β-cell functional genes and induction of genes characteristic of other islet cell types. It has been suggested that metabolic inflexibility is a key step of β-cell dedifferentiation and β-cell failure^2,11^. Interestingly, β-cell dedifferentiation and reprogramming appeared to be reversible upon normalization of glucose levels^12,13^. Recently, we have reported that β-cells are dedifferentiated in T2D patients with adequate glucose control and non-diabetic chronic pancreatitis, suggesting dedifferentiation can be a cause of β-cell failure, not merely as a consequence of hyperglycemia^14^. It still remains unclear whether certain signal pathway controls compromised β-cell identity, independent of hyperglycemia.

mTOR is an evolutionarily conserved, nutrient-sensing serine–threonine protein kinase, functioning in the form of at least two large protein complexes, mTOR complex 1 (mTORC1) and mTOR complex 2 (mTORC2)^15,16^. mTORC1 consists of RAPTOR (regulatory associated protein of mTOR), mLST8, PRAS40, DEPTOR, and mTOR, which is sensitive to Rapamycin^17,18^. Recent studies have shown that mTORC1 activity was upregulated in islets from db/db mice and human of T2D, indicating its critical role in adaptation and decompensation during diabetes progression^19,20^. The extensive research revealed that physiological mTORC1 activation is essential for β-cell development, growth, function, and survival^21,22^, whereas its sustained over-activation might lead to β-cell failure^23,24^. Recently, we have reported that β-cell specific *Raptor*-deficient mice disrupts postnatal β-cell growth and functional maturation, at least partly due to changes in DNA methylation pattern^25^. Later with the same knockout mice, Blandino-Rosano et al.^26^ found S6K signaling had more impact on β-cell size and apoptosis, while 4E-BP2/eIF4E activity was more involved in β-cell proliferation. However, the above observations were all collected in diabetic conditions, which made it obscure to which extent hyperglycemia per se underlies or exacerbates alterations in β-cell phenotype in mTORC1-deficient mice. Interestingly, in diabetic *Raptor*-KO islet, we observed decreased expression of β-cell identity genes and upregulation of progenitor markers. This work builds on our previous study, but takes it further by dissecting high glucose from gene deletion to explore the exact role of mTORC1 on β-cell identity and plasticity.

In the present study, we use 4-week-old β-cell lineage tracing *Raptor*-KO mice, keep their blood glucose at normal range for 4 weeks by implanting insulin pellets immediately after the onset of diabetes and then trace the fates of β-cells. Our results demonstrate that mTORC1 directly regulates adult β-cell identity, mitochondrial metabolic coupling, and protein synthetic activity to maintain mature β-cell differentiated phenotype. Moreover, *Raptor* is required for β-cell to suppress α-cell enriched genes, including α-cell transcription factor *MafB*, and a group of α-cell enriched genes, i.e. *Etv1*, *Tspan12* and thus prevent β- to α-cell reprograming at normal glucose range. Our data highlight mTORC1 signaling as an underlying mechanism implicated in promoting the terminal differentiation of β-cells and repressing β-cell default.

## Results

### Increased α/β-cell ratio in βRapKO^GFP^ mice

**Fig. 1:**
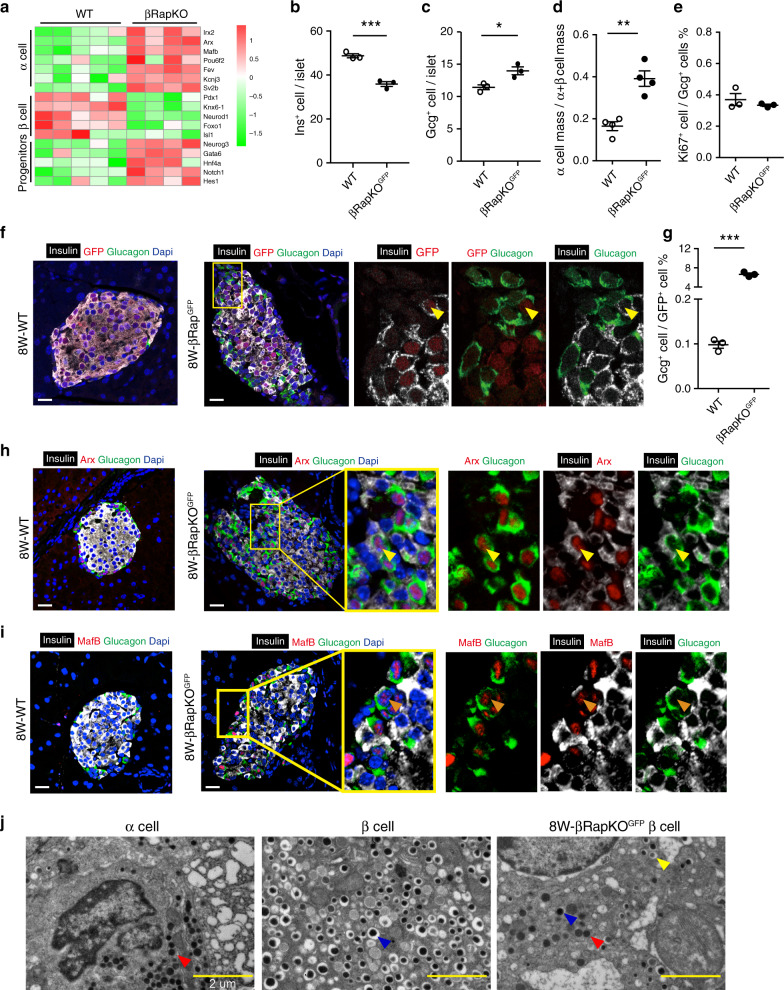
Increased α/β cell ratio in βRapKO^GFP^ mice. **a** Heatmap of genes critical to endocrine cell in wild type (WT) and βRapKO islets (*n* = 4–5). **b** The number of Ins^+^ cells per islet (*n* = 3, *p* = 0.0008) and **c** the number of Gcg^+^ cells per islet were calculated (*n* = 3, *p* = 0.023). At least, 46 islets were used for quantifications. **d** The proportion of pancreatic α-cell mass among pancreatic α- and β-cell mass was shown (*n* = 4, *p* = 0.0017). **e** The proliferation of Gcg^+^ cell was determined by quantification of the percentage of Ki67^+^ cells in Gcg^+^ cells (*n* = 3). At least, 50 islets were used for quantifications. **f** Pancreatic sections from 8-week-old WT, βRapKO^GFP^ mice were immunostained for insulin (white), glucagon (green), and GFP (red). Nuclei were stained with DAPI (blue) (*n* = 3). The yellow arrows indicated insulin, glucagon, and GFP co-stained cells. Scale bars, 20 μm. **g** The percentage of GFP^+^Gcg^+^ cells among GFP^+^ cells in 8-week-old WT and βRapKO^GFP^ mice (*n* = 3, *p* = 1.83046E-05). At least, 1837 GFP^+^ cells over three mice each group were used for quantifications. **h** Representative pancreatic sections showed expression of α-cells marker—Arx (red), insulin (white), and glucagon (green) in WT and βRapKO^GFP^ mice at 8 weeks of age (*n* = 3). Scale bars, 20 μm. **i** Immunostaining showed expression of α-cells marker—MafB (red), insulin (white), and glucagon (green) in control and βRapKO^GFP^ mice at 8 weeks of age (*n* = 3). Yellow boxes showed the specific areas of the islet, which were enlarged and represented by arrows on the right to demonstrate protein expression within specific cells. Scale bars, 20 μm. **j** Transmission electron microscopy (TEM) of pancreatic islets reveals a combination of mature insulin, immature insulin, and glucagon-like granules in the same cell in βRapKO^GFP^ mice (*n* = 3 independent samples). Granule identity is indicated by colored arrows: glucagon (red), mature insulin (blue), and immature insulin (yellow). Data represent means ± SEM. *p < 0.05, **p < 0.01, ***p < 0.001 by two-sided Student’s *t-*test.

Recently, we have reported that *Raptor* regulates functional maturation in murine β-cells^25^. The heatmap showed that loss of *Raptor* reduced the expressions of genes critical to β-cell (*Pdx1, Nkx6.1, NeuroD1, Foxo1, Isl1)*, whereas the expressions of α-cell key factors (*Irx2, Arx, MafB, Pou6f2, Fev, Kcnj3*, and *Sv2b*) and progenitor markers (*Neurog3, Gata6, Hnf4a, Notch1*, and *Hes1*) (Fig. 1a) were strongly upregulated in mutant islets. These data suggest that *Raptor*-deficient immature β-cells fail to maintain β-cell identity and adopt progenitor-like features with the tendency to convert to α-cells.

To elucidate the role of mTORC1 in maintaining β-cell identity, we deleted *Raptor* which is an essential and specific component of mTORC1 in β-cells and traced their fates using a *Rosa26*^*GFP*^ lineage labeling. This was achieved by generating *RIP-Cre; Raptor*^*−/−*^*; Rosa26*^*GFP*^ (βRapKO^GFP^) mice and their control littermates *RIP-Cre; Raptor*^*+/+*^*; Rosa26*^*GFP*^ (WT) (Supplementary Fig. 1a). GFP expression was exclusively detected in the insulin-producing cells in the pancreas of *RIPCre-Rosa26*^*GFP*^ mice (Supplementary Fig. 1b) and GFP^+^ cells can be obtained by fluorescence-activated cell sorting (FACS) (Supplementary Fig. 1c). The *Raptor* mRNA level was almost undetectable in β-cells but was abundantly expressed in other tissues such as heart, kidney, muscle, liver, and hypothalamus (Supplementary Fig. 1d). The islets isolated from βRapKO^GFP^ mice showed reduced expression of RAPTOR and de-phosphorylation of mTORC1 targets PS6 (Ser240/244) and 4E-BP1 (shift from the highly phosphorylated γ-band to the non-phosphorylated α-band and an intermediate β-band) (Supplementary Fig. 1e). Moreover, loss of mTORC1 activity (PS6 Ser240/244) could only be detected in insulin-positive (Ins^+^) cells of dispersed mutant islets (Supplementary Fig. 1f).

βRapKO^GFP^ mice started to display elevated random and 6 h fasting blood glucose levels at the age of 4 weeks (Supplementary Fig. 2a, b), and they developed overt diabetes at the age of 8 weeks when challenged with intraperitoneal glucose injection (Supplementary Fig. 2c). The diabetic phenotype was in line with our previous observations on βRapKO mice^25^. We found approximately 70% reduction in 6 h fasting plasma insulin levels (Supplementary Fig. 2d), but not in 6 h fasting glucagon concentrations (Supplementary Fig. 2e) in 8-week-old βRapKO^GFP^ mice. Accordingly, the Ins^+^ cells per islet (Fig. 1b) and β-cell mass (Supplementary Fig. 2f) were significantly reduced in βRapKO^GFP^ mice. Importantly, we detected that Gcg^+^ cells per islet were significantly increased (13.98 ± 0.61 vs 11.43 ± 0.37 in WT, *p*  < 0.05, Fig. 1c) in βRapKO^GFP^ islets. Although the α-cell mass only tended to increase (Supplementary Fig. 2g), the α-cell mass/(α + β)-cell mass was doubled in βRapKO^GFP^ mice (Fig. 1d). Interestingly, the increased α-cells were not derived from the proliferation of themselves, as quantitative analysis revealed that the percentage of Ki67^+^/Gcg^+^ cells remained unaltered in mutant islets (Fig. 1e). At 12 weeks of age, further decreases in Ins^+^ cells (43.23 ± 3.36 vs 57.01 ± 3.48 in WT, *p* < 0.05) and increases in Gcg^+^ cells (22.04 ± 1.72 vs 13.35 ± 0.69 in WT, *p* < 0.05) were detected in βRapKO^GFP^ islets (Supplementary Fig. 2h).

We then tested whether there existed β to α shift upon loss of *Raptor*. We performed co-immunostaining for GFP, insulin, and glucagon on pancreatic sections from 8-week-old WT and βRapKO^GFP^ mice. We found significantly increased number of *Raptor*-deficient GFP cells coexpressing glucagon in mutant islets (6.65% ± 0.27 vs 0.10% ± 0.008 in WT, *p* < 0.001) (Fig. 1f, g). However, we did not find evidence of β-cell reprogramming to δ or PP cells: neither somatostatin nor pancreatic polypeptide was co-localized with GFP/Ins^+^ in βRapKO^GFP^ islet (Supplementary Fig. 2i, j). These results suggest that *Raptor*-deficient β-cells lose their identities and adopt α-cell fates.

If β-cells were the source of newly formed α-cells, we hypothesized that, during the transition phase, the transcription factors crucial for α-cell might be expressed in these multi-hormonal GFP cells. Indeed, we detected the expression of α-cell specific transcription factors Arx (Fig. 1h, Supplementary Fig. 3a) and/or MafB (Fig. 1i, Supplementary Fig. 3b) in Ins^+^Gcg^+^ cells in 8-week-old βRapKO^GFP^ islets. Moreover, the multi-hormonal cells included both Arx^+^ and Arx^−^ subpopulations (Supplementary Fig. 3c), representing different converted stages during transition. Interestingly, we also identified expressions of Glut2 (Supplementary Fig. 3d) and/or Pdx1 (Supplementary Fig. 3e) in some Ins^+^Gcg^+^ cells, indicating some multi-hormonal cells retained expression of β-cell crucial genes. These “mixed phenotype” cells provide evidence that *Raptor* knockout β-cells achieve α-like features.

Electron microscopy was also performed on 8-week-old WT and βRapKO^GFP^ islets. The light microscopy showed that intact WT mature β-cells display typical insulin granules with characteristic electron-dense insulin crystal cores surrounded by a clear “halo” (Fig. 1j, middle panel, blue arrow), whereas glucagon-containing granules in α-cells lack any such “halo” (Fig. 1j, left panel, red arrow). In contrast, we observed a few *Raptor*-deficient β-cells had mixed features of α- and β-granules, some with dark (mature insulin granule, Fig. 1j, right panel, blue arrow) or light electron-dense crystal cores (immature insulin granule, Fig. 1j, right panel, yellow arrow) and others lost their outer “halo” (Fig. 1j, right panel, red arrow). These findings further support that *Raptor*-deficient β-cells have morphological features of α-like cells.

### Impaired proinsulin synthesis and ATP levels for GSIS

**Fig. 2:**
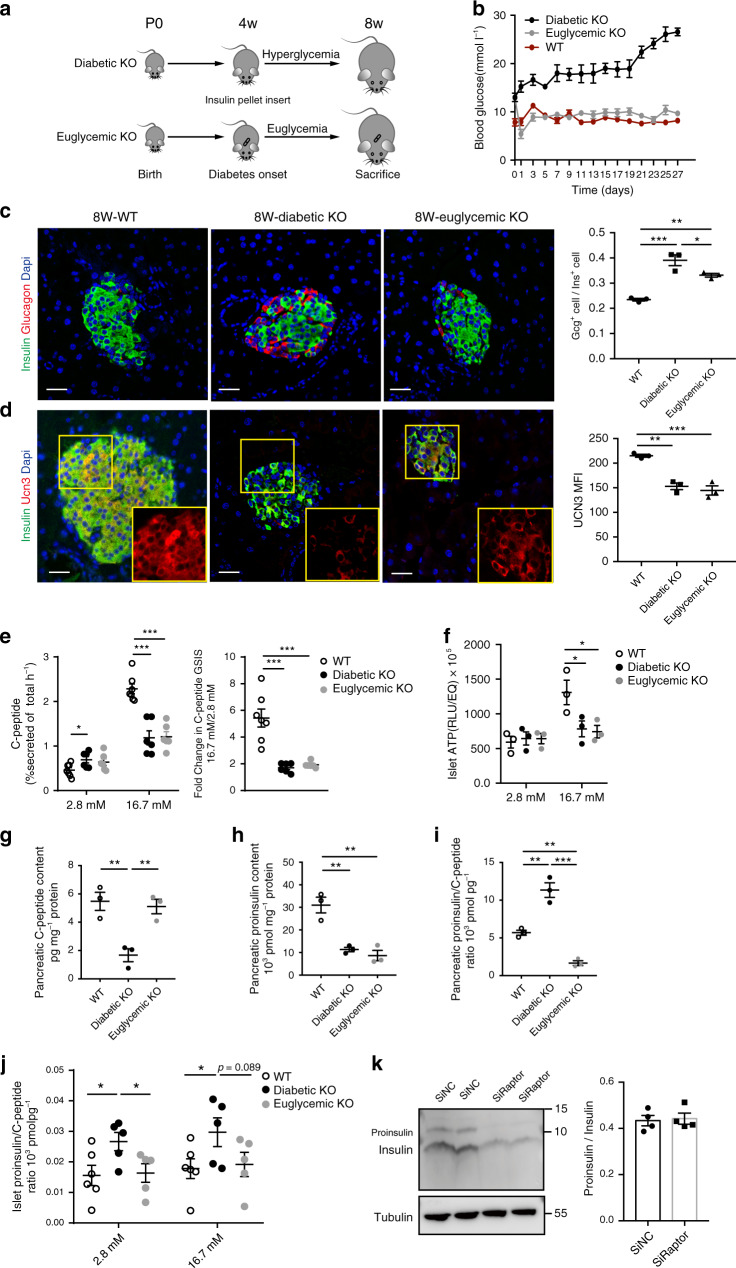
Impaired proinsulin synthesis and ATP levels for GSIS. **a** Schematic of insulin pump implantation. **b** Random blood glucose levels were monitored every other day in WT, diabetic βRapKO^GFP^, and euglycemic βRapKO^GFP^ mice from 4 weeks to 8 weeks old (*n* = 6 for WT, *n* = 7 for diabetic KO, *n* = 13 for euglycemic KO). **c** Representative pancreatic sections immunostained for insulin (green) and glucagon (red). The ratio of Gcg^+^ cells to Ins^+^ cells was calculated (*n* = 3). At least 50 islets or 2000 insulin-positive cells were used for quantifications. Scale bars, 20 μm. **d** Images of islets labeled with insulin (green) and UCN3 (red). MFI of UCN3 in these three groups (*n* = 3). At least 10 islets from three sections were used for MFI. Yellow boxes showed the expression of UCN3. Scale bars, 20 μm. **e** In vitro glucose-stimulated C-peptide secretion in islets at 2.8 and 16.7 mM glucose levels for 1 h (reported as percent of C-peptide content) (*n* = 7 for WT, *n* = 6 for diabetic KO, *n* = 6 for euglycemic KO). White, black, and gray circles represented the WT, diabetic βRapKO^GFP^, and euglycemic βRapKO^GFP^ groups, respectively. Stimulation index of C-peptide secretion in diabetic βRapKO^GFP^ and euglycemic βRapKO^GFP^ mice as compared with controls (*n* = 7 for WT, *n* = 6 for diabetic KO, *n* = 6 for euglycemic KO). **f** ATP content at 2.8 mM glucose and 16.7 mM glucose (*n* = 3). **g** Pancreatic C-peptide content (*n* = 3 independent samples) and **h** pancreatic proinsulin content normalized by protein concentration were shown (*n* = 3 independent samples). **i** The ratio of pancreatic proinsulin to C-peptide content was determined (*n* = 3 independent samples). **j** The ratio of islet proinsulin to C-peptide content per 10 size-matched islets at 2.8 mM and high 16.7 mM glucose levels was determined (*n* = 6 for WT, *n* = 5 for diabetic KO, *n* = 5 for euglycemic KO). **k** INS-1 cells were transfected with Si*Raptor* or SiNC for 72 h. Immunoblotting and quantification of insulin and proinsulin in INS-1 cells transfected with SiNC or Si*Raptor* (*n* = 4 independent cell experiments). Data represent means ± SEM. *p < 0.05, **p < 0.01, ***p < 0.001 by two-sided Student’s *t-*test and one-way ANOVA adjusted by LSD multiple comparison, *p* values included in source data.

The occurrence of hyperglycemia in diabetes can diminish β-cell function and alter β-cell identity^27^. To determine the independent impact of *Raptor* and hyperglycemia on β-cell identity and function, we implanted slow-release insulin pellet on 4-week-old βRapKO^GFP^ mice (the age when fasting blood glucose levels started to rise) for 4 weeks and kept the serum blood glucose at normal levels in mutant rodents (Fig. 2a). As expected, implantation of insulin pellet (releasing 0.2–0.3 U per day) caused a rapid fall in random blood glucose from 12.86 ± 0.37 to 5.43 ± 0.96 mM on the day of implantation, 2 days later to 8.92 ± 0.80 mM (Fig. 2b). Afterwards, insulin-treated βRapKO^GFP^ mice (euglycemic βRapKO^GFP^) maintained normoglycemia for 4 weeks, with comparable blood glucose levels as that of WT mice, whereas untreated mutant mice (diabetic βRapKO^GFP^) exhibited severe hyperglycemia (Fig. 2b). Insulin treatment for 4 weeks partially prevented the change in islet morphology (Fig. 2c) and restored MafA expression in mutant mice (Supplementary Fig. 4a). On the contrary, the severely reduced expression levels of maturity marker UCN3 observed in diabetic βRapKO^GFP^ islet^25^ (Fig. 2d) were not improved in euglycemic mutants (Fig. 2d). It is known that Ucn3 is exclusively expressed in mature β-cells and is required for full glucose-stimulated insulin secretion in mouse islet^28,29^. We then performed GSIS in isolated islets from the three groups and measured C-peptide levels to obviate assessment of exogenous insulin. Compared to WT, both diabetic and euglycemic βRapKO^GFP^ islets exhibited increased glucose threshold for C-peptide secretion: i.e. elevated basal C-peptide secretion, but significantly diminished glucose-stimulated C-peptide secretion at 16.7 mM glucose (Fig. 2e). The diminished stimulation index (fold change in GSIS) in euglycemic mutant islet unveiled that *Raptor* itself was the cause of impaired C-peptide secretory response to glucose (Fig. 2e). In parallel, the islet ATP content was comparable at basal glucose concentrations among the three groups, whereas acute glucose-stimulated ATP increase was significantly reduced (*p* < 0.05) in both diabetic and euglycemic βRapKO^GFP^ mice (Fig. 2f).

Intriguingly, though pancreatic C-peptide content in euglycemic βRapKO^GFP^ was completely restored (Fig. 2g), pancreatic proinsulin content was still markedly reduced compared to WT (Fig. 2h), indicating impaired proinsulin biosynthetic activity in β-cell is the main defect due to loss of *Raptor*. It has previously been suggested that *Raptor* deficiency impairs proinsulin processing by impairing the synthesis of Carboxypeptidase E (CPE)^26^. We found the proinsulin/C-peptide ratio of both pancreatic tissue (Fig. 2i) and cultured islets (at 2.8 and 16.7 mM glucose) (Fig. 2j) were significantly increased in diabetic βRapKO^GFP^ mice, but were completely reversed in euglycemic βRapKO^GFP^ mice. We further performed double staining of insulin and CPE on 8- and 2-week-old mutant pancreas. Interestingly, we found that the expression of CPE was decreased in diabetic βRapKO^GFP^ mice, while in euglycemic βRapKO^GFP^ mice the expression of CPE was reversed (Supplementary Fig. 4b). Moreover, in 2-week-old euglycemic βRapKO^GFP^ mice, CPE expression was comparable to that of WT controls (Supplementary Fig. 4c). To further test the specific effect of mTORC1 on CPE expression and proinsulin/insulin ratio, we silenced *Raptor* in INS-1 cells. Knockdown of *Raptor* dramatically reduced both proinsulin and insulin abundance shown in immunoblot, but presented a comparable proinsulin/insulin ratio in these two groups (Fig. 2k). Moreover, we did not detect difference in mRNA expression of processing genes (*Pcsk1*, *Pcsk2*, and *CPE*) (Supplementary Fig. 4d) and protein abundance of CPE (Supplementary Fig. 4e) between SiNC and Si*Raptor* conditions. Taken together, our data demonstrate that loss of *Raptor* had detrimental impacts on proinsulin production, metabolic coupling of insulin secretion in β-cells, but the abnormal increase in proinsulin/insulin ratio and defects in CPE expression were the consequences of hyperglycemia.

### Impaired metabolic coupling of insulin secretion

**Fig. 3:**
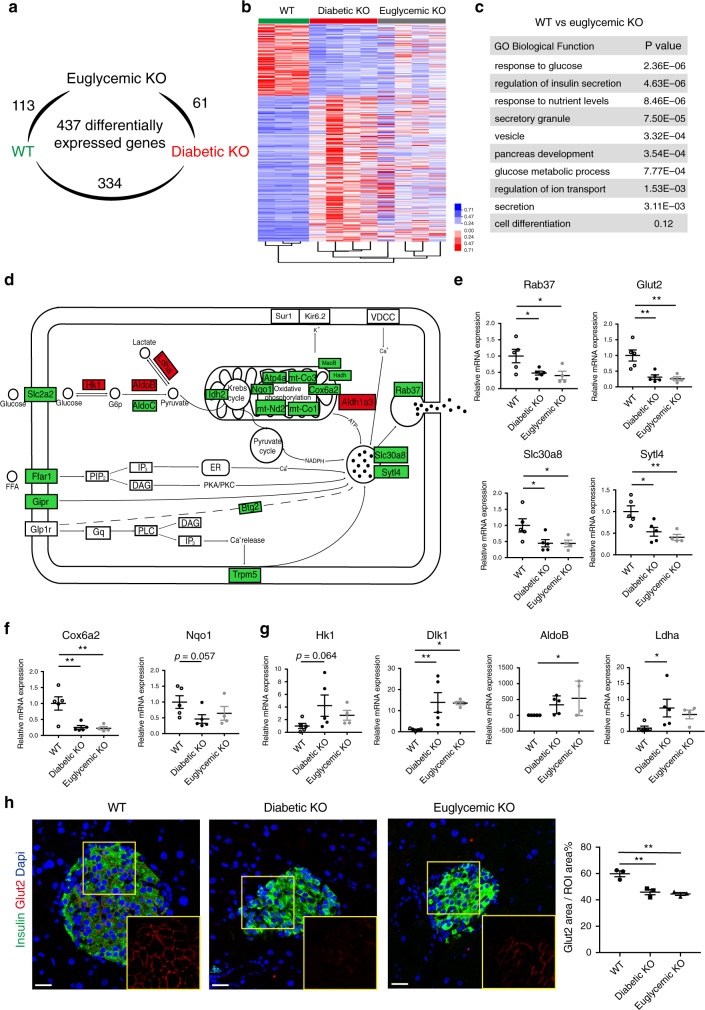
Impaired metabolic coupling of insulin secretion. **a** The number of differentially expressed genes within the three groups (*n* = 3–4). **b** Gene expression profiles regulated by *Raptor* were subjected to hierarchical clustering (*n* = 3–4). **c** GO analysis of differentially expressed genes as identified by RNA-seq of 8-week-old WT (*n* = 3) and euglycemic βRapKO^GFP^ β-cells (*n* = 4) was associated with β-cell function. **d** Visualization of differential expression of genes involved in insulin secretion. **e–g** Relative expression of selected transcripts associated with β-cell secretion function (*n* = 5 independent sample for WT, *n* = 5 independent sample for diabetic βRapKO^GFP^, *n* = 4 independent sample for euglycemic βRapKO^GFP^, *p* values included in source data) (**e**), mitochondrial metabolism (*n* = 5 independent sample for WT, *n* = 5 independent sample for diabetic βRapKO^GFP^, *n* = 4 independent sample for euglycemic βRapKO^GFP^, *p* values included in source data) (**f**), and disallowed genes (*n* = 5 independent sample for WT, *n* = 5 independent sample for diabetic βRapKO^GFP^, *n* = 4 independent sample for euglycemic βRapKO^GFP^, *p* values included in source data) (**g**) in WT, diabetic βRapKO^GFP^, and euglycemic βRapKO^GFP^ β-cells by qRT-PCR. **h** Representative immunofluorescent staining for Glut2 (red) and insulin (green) among 8-week-old WT, diabetic βRapKO^GFP^, and euglycemic βRapKO^GFP^ mice (*n* = 3). Insets showed different expression levels of Glut2. Quantification of Glut2 areas in Ins^+^ areas in WT, diabetic βRapKO^GFP^, and euglycemic βRapKO^GFP^ mice (*n* = 3, WT vs diabetic βRapKO^GFP^: *p* = 0.002; diabetic βRapKO^GFP^ vs euglycemic βRapKO^GFP^: *p* = 0.613; WT vs euglycemic βRapKO^GFP^: *p* = 0.001), at least 10 islets from three sections were used for quantifications of Glut2. Scale bars, 20 μm. Data represent means ± SEM. *p < 0.05, **p < 0.01, ***p < 0.001 by one-way ANOVA.

To identify *Raptor-*regulated pathways in β-cells, we conducted RNA-sequencing analysis on FACS-sorted GFP^+^ cells obtained from 8-week-old WT, diabetic, and euglycemic βRapKO^GFP^ mice. After filtering out the repeated genes, we obtained a list of 437 differentially expressed genes ≥1.5-fold change and with *p* < 0.05 for at least one of the possible comparisons between two groups. Within these 437 genes, the number of differentially expressed genes for WT vs diabetic βRapKO^GFP^ was 334, WT vs euglycemic βRapKO^GFP^ was 113, and diabetic βRapKO^GFP^ vs euglycemic βRapKO^GFP^ was 61 (Fig. 3a). We then subjected the differentially expressed genes regulated by *Raptor* to hierarchical clustering and found that diabetic and euglycemic βRapKO^GFP^ groups were most closely clustered, whereas diabetic/euglycemic βRapKO^GFP^ and WT were distantly clustered (Fig. 3b). The limited number of changed genes and closely clustering between diabetic and euglycemic βRapKO^GFP^ groups indicate that hyperglycemia played a relatively mild influence on the main phenotype. To identify the changes directly resulted from *Raptor* deletion, we performed Gene Ontology analysis on differentially expressed genes between WT and euglycemic βRapKO^GFP^ β-cells. The analysis showed that *Raptor* preferentially regulated transcripts critical for β-cell function, i.e. response to glucose, insulin secretion, glucose metabolic process, secretory granule, vesicle/ion transport, and pancreas development (Fig. 3c).

The transcriptome changes from the RNA-seq encompassed virtually key pathways required for physiologic insulin secretion, which allowed us to formulate a hypothesis on the physiologic role of *Raptor* in β-cells (Fig. 3d). The genes required for fuel metabolism (*Glut2*, *Idh2*, *Atp4a*, *Cox6a2*, *mt-Co3*, *mt-Co1*, *mt-Nd2*, *Nqo1*, *Ffar1*) were all significantly downregulated in both diabetic and euglycemic *Raptor*-deficient β-cells (Fig. 3d). In addition to mitochondrial dysfunction, loss of *Raptor* as previously reported^25^ caused upregulation of disallowed genes *Ldha*, *Hk1*, and *AldoB*, which could not be rescued after glucose normalization (Fig. 3d). Moreover, secretory pathway (*Gipr*, *Trpm5*, *Btg2*, *MaoB*, *Fkbp1b*) and granule trafficking (*Slc30a8*, *Sytl4*, *Rab37*) were obviously decreased in both diabetic and euglycemic *Raptor*-deficient β-cells (Fig. 3d). On the contrary, some genes involved in anti-inflammatory (*Dusp1*, *Hcar2*), eNOS, and ROS (*Pecam1*, *Arg1*, *CEPBδ*, *Eng*, and *Αqp11)* process were completely reversed in euglycemic βRapKO^GFP^ β-cells, indicating that inflammatory and ROS pathway were induced by glucotoxicity rather than loss of *Raptor*.

We confirmed dramatic decreases in glucose transporter *Glut2*, subunit of the cytochrome *c* oxidase complex *Cox6a2*, member of NAD(P)H dehydrogenase (quinone) family *Nqo1* in β-cells from both diabetic and euglycemic βRapKO^GFP^ mice using qRT-PCR (Fig. 3e–f). Moreover, the expression levels of *Slc30a8* (*ZnT8*) required for insulin crystallization/secretion and *Sytl4*, *Rab37* participating in insulin granule trafficking and docking were all decreased significantly in both diabetic and euglycemic βRapKO^GFP^ β-cells (Fig. 3e). The ectopically upregulation of disallowed genes *Hk1*, *AldoB*, and *Ldha* in euglycemic βRapKO^GFP^ β-cells were reproduced by qRT-PCR (Fig. 3g). We further compared our RNA-seq data with the disallowed gene list from Pullen et al.^30^, and found many disallowed genes (i.e. *Serpina7*, *Gm2115*, *Gucy2c*, *Aass*, *Trf*, *Hmgcs2*, *Gfra1*, *Ptprk*, *Tspan12*, and *Sh3bgrl2*) were upregulated in *Raptor*-deficient β-cells (Supplementary Fig. 5). The immunostaining experiment identified that GLUT2 protein expression was significantly decreased in βRapKO^GFP^ islet, and this dramatic change was not reversed in euglycemic mutants (Fig. 3h).

### Induction of α-cell-enriched genes in euglycemic βRapKO^GFP^

**Fig. 4:**
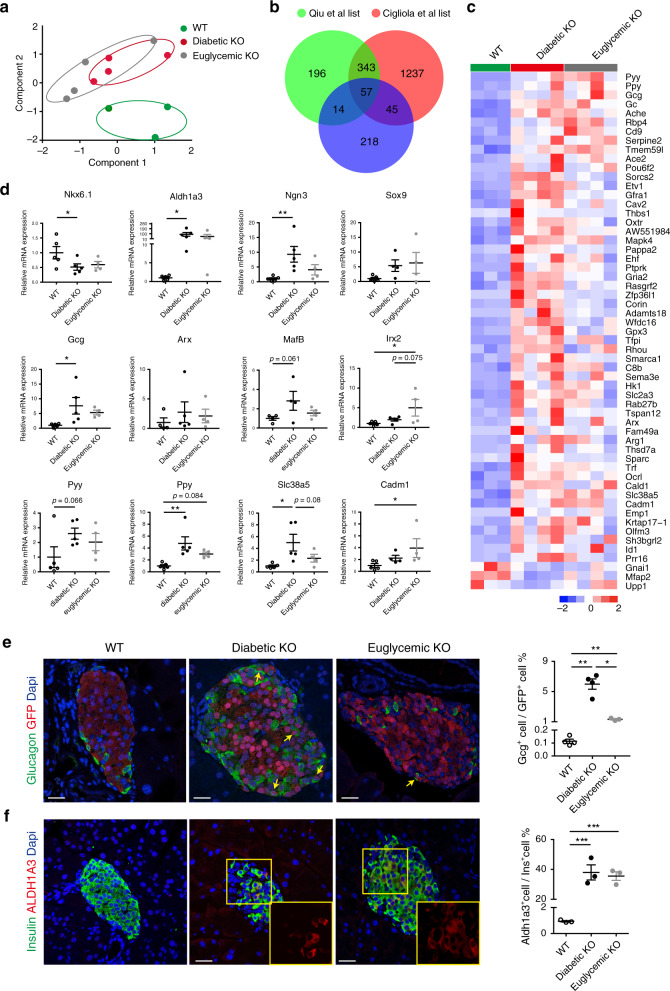
Induction of α-cell-enriched genes in euglycemic βRapKO^GFP^. **a** α-Cell-enriched genes and β-cell-enriched genes were subjected to perform principle component analysis based on the dataset from Qiu et al.^31^. **b** Venn diagram representation of the subsets of *Raptor*-regulated genes that were enriched in α-cells. **c** Heatmap showed the differential expression of 57 overlapped genes in 8-week-old WT, diabetic βRapKO^GFP^, and euglycemic βRapKO^GFP^ β-cells (*n* = 3-4). **d** Relative expression of genes involved in cell identity by qRT-PCR (*n* = 5 independent sample for WT, *n* = 5 independent sample for diabetic βRapKO^GFP^, *n* = 4 independent sample for euglycemic βRapKO^GFP^, *p* values included in source data). **e** Representative immunofluorescent staining for GFP (red) and glucagon (green) among 8-week-old WT, diabetic βRapKO^GFP^, and euglycemic βRapKO^GFP^ mice (*n* = 4 for WT, *n* = 4 for diabetic βRapKO^GFP^, *n* = 3 for euglycemic βRapKO^GFP^). The yellow arrows indicated glucagon and GFP co-stained cells. Percentage of Gcg^+^GFP^+^ cells among GFP^+^ cells in WT, diabetic βRapKO^GFP^, and euglycemic βRapKO^GFP^ mice was determined (*n* = 4 independent sample for WT, *n* = 4 independent sample for diabetic βRapKO^GFP^, *n* = 3 independent sample for euglycemic βRapKO^GFP^, WT vs diabetic βRapKO^GFP^: *p* = 0.007; diabetic βRapKO^GFP^ vs euglycemic βRapKO^GFP^: *p* = 0.012; WT vs euglycemic βRapKO^GFP^: *p* = 0.009). At least 50 islets were used for quantifications. Scale bars, 20 μm. **f** Representative immunofluorescent staining for ALDH1A3 (red) and insulin (green) among 8-week-old WT, diabetic βRapKO^GFP^, and euglycemic βRapKO^GFP^ mice (*n* = 3). Insets showed different expression levels of ALDH1A3. Percentage of ALDH1A3^+^Ins^+^ cells among Ins^+^ cells in WT, diabetic βRapKO^GFP^, and euglycemic βRapKO^GFP^ mice was calculated (*n* = 3, WT vs diabetic βRapKO^GFP^: *p* < 0.001; diabetic βRapKO^GFP^ vs euglycemic βRapKO^GFP^: *p* = 0.628; WT vs euglycemic βRapKO^GFP^: *p* < 0.001). For ALDH1A3 quantification, at least 36 islets were used. Scale bars, 20 μm. Data represent means ± SEM. *p < 0.05, **p < 0.01, ***p < 0.001 by one-way ANOVA.

To get a better understanding whether alterations in β-cell identity and plasticity in *Raptor*-deficient β-cells were reversible upon normalization of blood glucose levels, we performed the principal component analysis (PCA) based on two published datasets of signature genes individually enriched in β- and α-cells^31,32^. Notably, in the PCA plot, diabetic and euglycemic βRapKO^GFP^ groups were most closely clustered, whereas diabetic/euglycemic βRapKO^GFP^ and WT were distantly clustered (Fig. 4a). Based on the reference lists by Qiu et al.^31^ and Cigliola et al.^32^, in purified GFP-sorted β-cells, *Raptor* regulated a subset of genes (57 genes) that are highly expressed in α-cell lineage, among which 94.7% were significantly upregulated (Figs. 4b,c). Importantly, many of these α-cell-enriched genes maintained at high expression levels in euglycemic βRapKO^GFP^ β-cells (Fig. 4c). By qRT-PCR, we demonstrated that important β-cell identity genes, *Nkx6.1* and *Pdx1* were significantly downregulated, whereas progenitor markers such as *Aldh1a3*, *Ngn3*, *Sox9* were upregulated in both diabetic and euglycemic βRapKO^GFP^ β-cells (Fig. 4d, Supplementary Fig. 6a). Moreover, the important α-cell gene product *glucagon* and crucial α-cell transcription factors *MafB* and *Irx2* were significantly upregulated in diabetic βRapKO^GFP^ β-cells and remained at high expression levels in euglycemic βRapKO^GFP^ β-cells (Fig. 4d). Another α-cell transcription factor *Arx* tended to increase in the βRapKO^GFP^ group but did not reach statistical significance (Fig. 4d). In addition, several genes important for α-cell, i.e. *Pyy*, *Ppy*, *Slc38a5*, *Cadm1*, and *Ace2* were all significantly upregulated in purified β-cells from euglycemic βRapKO^GFP^ mice (Fig. 4d, Supplementary Fig. 6a). These results indicate that *Raptor* determines β-cell identity and plasticity, independent of hyperglycemia.

We then measured GFP^+^ multi-hormonal cells in the three groups. The percentage of GFP^+^Gcg^+^ cells among all GFP^+^ cells in diabetic βRapKO^GFP^ were significantly increased (5.99% ± 0.69 vs 0.11% ± 0.02 in WT, *p* < 0.01). The multi-hormonal cells were much less in euglycemic mutants but still remained at a 13-fold increase compared to WT (1.32% ± 0.09 vs 0.11% ± 0.02 in WT, *p* < 0.01) (Fig. 4e). We further applied in situ immunostaining against ALDH1A3, a β-cell dedifferentiation marker, on pancreatic section of the three groups. ALDH1A3 was strongly induced in Ins^+^ cells of diabetic βRapKO^GFP^ mice (37.95% ± 5.06 vs 0.94% ± 0.06 in WT, *P* < 0.05) and the high proportion of Aldh1a3^+^Ins^+^ cells remained (35.52% ± 2.88) in euglycemic βRapKO^GFP^ mice when glycemia has been adequately controlled (Fig. 4f). In addition, we found that some β-cells from euglycemic βRapKO^GFP^ mice still lost β-cell specific transcriptional factor Nkx6.1 (Supplementary Fig. 6b). Collectively, these results indicate that *Raptor* itself orchestrates many of transcriptional changes that define fate map for β-cell before the onset of hyperglycemia, while hyperglycemia accelerates the final decision.

### mTORC1 regulates α-cell transcriptional factor MafB

**Fig. 5:**
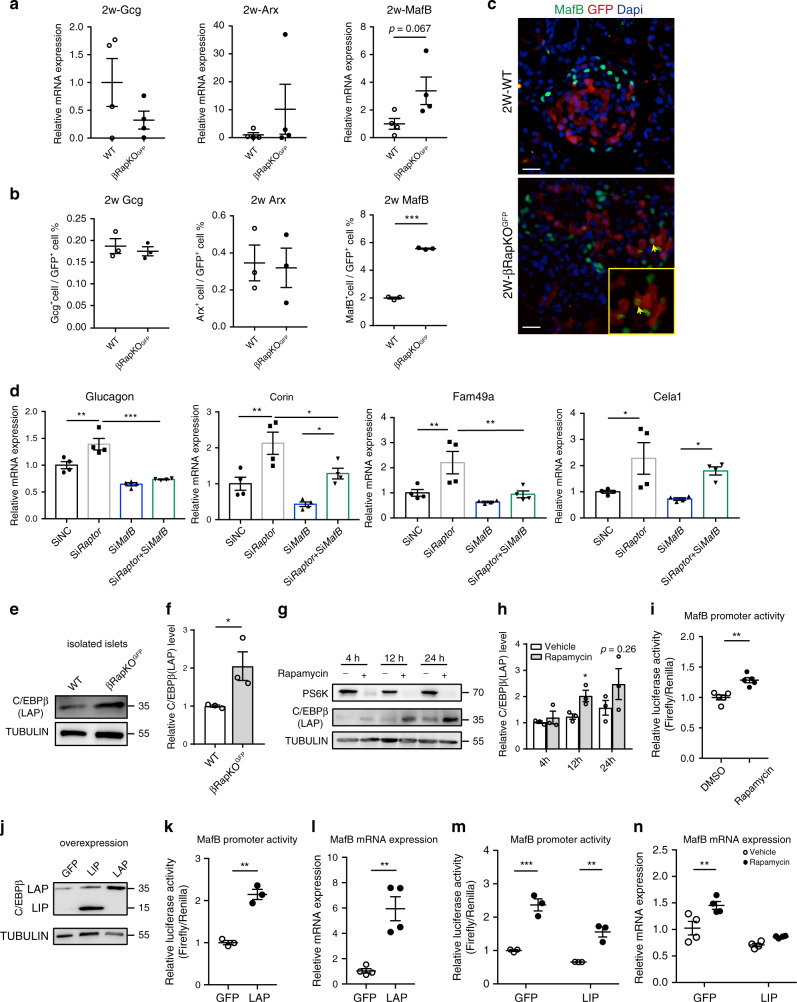
mTORC1 regulates α-cell transcriptional factor MafB. **a** Relative expression of *glucagon*, *Arx,* and *MafB* in 2-week-old WT and βRapKO^GFP^ β-cells by qRT-PCR (*n* = 4). **b** Quantification of Gcg^+^GFP^+^, Arx^+^GFP^+^, and MafB^+^GFP^+^ cells in GFP^+^ cells in 2-week-old WT and βRapKO^GFP^ mice (*n* = 3, Gcg: *p* = 0.58; Arx: *p* = 0.86; MafB: *p* = 9.00122E-07). At least 1783 GFP^+^ cells were used for quantifications. c Representative immunofluorescent staining for MafB (green) and GFP (red) in 2-week-old WT and βRapKO^GFP^ mice (*n* = 3). Yellow box showed the specific area of the islet, which was enlarged and represented by arrows to demonstrate MafB^+^GFP^+^ cell. Scale bars, 20 μm. **d** Relative expression of selected transcripts associated with α-cell-enriched genes in INS-1 cells which were transfected with SiNC or Si*Raptor* in the presence or absence of Si*MafB* by qRT-PCR (*n* = 4 independent cell experiments, *p* values included in source data). **e**, **f** Western blotting showed the expression of C/EBPβ(LAP) in 8-week-old WT and βRapKO^GFP^ mice (*n* = 3, *p* = 0.049). **g**, **h** INS-1 cells were treated with Rapamycin (25 nM), C/EBPβ(LAP) protein expression was assayed by immunoblot (*n* = 3 independent cell experiments). **i** Luciferase reporter gene assays revealed that Rapamycin treatment for 12 h positively regulated the luciferase activity of MafB (*n* = 5 independent cell experiments, *p* = 0.0011). **j** Immunoblotting to evaluate the LAP and LIP protein levels after LAP and LIP overexpression (*n* = 2 independent cell experiments). **k** Luciferase reporter assay using a rat MafB promoter reporter. INS-1 cells were transfected with GFP or LAP overexpression vector (*n* = 3 independent cell experiments, *p* = 0.001). **l** qRT-PCR analysis of *MafB* expression in GFP and LAP overexpressed INS-1 cells (*n* = 4 independent cell experiments, *p* = 0.0023). **m** Luciferase reporter assay using a rat MafB promoter reporter. INS-1 cells were transfected with GFP or LIP overexpression vector in the absence and presence of Rapamycin (*n* = 3 independent cell experiments, GFP + Vehicle vs GFP + Rapamycin, *p* < 0.001; LIP + Vehicle vs LIP + Rapamycin, *p* = 0.001). **n** qRT-PCR analysis of *MafB* expression in GFP and LIP overexpressed INS-1 cells in the absence or presence of Rapamycin (*n* = 4 independent cell experiments, GFP + Vehicle vs GFP + Rapamycin, *p* = 0.002; LIP + Vehicle vs LIP + Rapamycin, *p* = 0.177). Data represent means ± SEM. *p < 0.05, **p < 0.01, ***p < 0.001 by two-sided Student’s *t-*test and one-way ANOVA.

We then assessed essential α-cell transcriptional factors and glucagon in purified β-cells from 2-week-old βRapKO^GFP^ mice and their age-matched WT mice. At 2 weeks of age, βRapKO^GFP^ mice showed normal glycemic levels (Supplementary Fig. 2a) and intact islet structure. Interestingly, we detected a dramatic induction of *MafB*, but not *Arx* and/or *glucagon* in purified mutant β-cells by qRT-PCR (Fig. 5a). We then performed immunostaining for GFP and glucagon/Arx/MafB on pancreatic sections from 2-week-old WT and βRapKO^GFP^ mice. We did not detect changes in the proportion of GFP^+^Gcg^+^ or GFP^+^Arx^+^ cells between the two groups, but a significant increase in the percentage of GFP^+^ cells coexpressing MafB was readily detected in mutant islets (5.56% ± 0.04 vs 1.99% ± 0.07 in WT, *p* < 0.001) (Fig. 5b, c). This observation was consistent with our previous finding in islet from βRapKO^25^. It is known that MafB binds to and directly activates glucagon gene expression in α-cells^33^. In order to determine whether transcriptional factor *MafB* can regulate *glucagon* expression and other α-cell enriched genes, we chose *Glucagon*^33^ and three other α-cell enriched genes *(Corin*, *Fam49a*, *Cela1*) based on MafB open chip-seq database^34,35^ for rescue experiments in INS-1 cells. Interestingly, Si*Raptor* significantly upregulated *Glucagon*, *Corin*, *Fam49a*, and *Cela1* expression, which can be completely or partially reversed by silencing *MafB* expression (Fig. 5d, Supplementary Fig. 7). Taken together, *Raptor* is required for repressing *MafB* and other α-cell signature gene before the onset of hyperglycemia.

It is known that the isoform ratio of C/EBPβ directs cell differentiation^36^ and mTORC1 can regulate MafB expression by modulating the C/EBPβ isoform ratio in monocytes^37^. Interestingly, we found the protein level of LAP, an isoform of C/EBPβ, was significantly increased in isolated islets from βRapKO^GFP^ mice (Fig. 5e, f). To further confirm whether mTORC1 directly regulates C/EBPβ and MafB expression, we treated INS-1 cells with mTORC1 inhibitor Rapamycin. Rapamycin treatment for 4 h dramatically decreased mTORC1 target PS6K expression, later at 12 h significantly increased C/EBPβ LAP expression and enhanced MafB promoter activity in INS-1 cells (Fig. 5g–i).

We then tested whether the effect of mTORC1 on MafB expression was via C/EBPβ. We overexpressed the C/EBPβ isoform LAP in INS-1 cells as a transcriptional activator (Fig. 5j), and C/EBPβ LAP overexpression was found to increase both MafB promoter activity (Fig. 5k) and *MafB* mRNA expression (Fig. 5l). The truncated C/EBPβ isoform LIP lacked the N-terminal transactivation domains but still possessed the DNA-binding domain, thus can act as a competitive inhibitor of LAP function^37^. Importantly, C/EBPβ LIP overexpression could significantly reverse Rapamycin-induced activation in MafB promoter activity and in *MafB* mRNA expression (Fig. 5m, n). These data suggest that loss of *Raptor* shifts C/EBPβ to LAP isoform, which directly upregulates MafB expression and possibly contributes to multi-hormonal cell fate.

### Induction of α-cell-enriched genes in 2-week-old βRapKO^GFP^

**Fig. 6:**
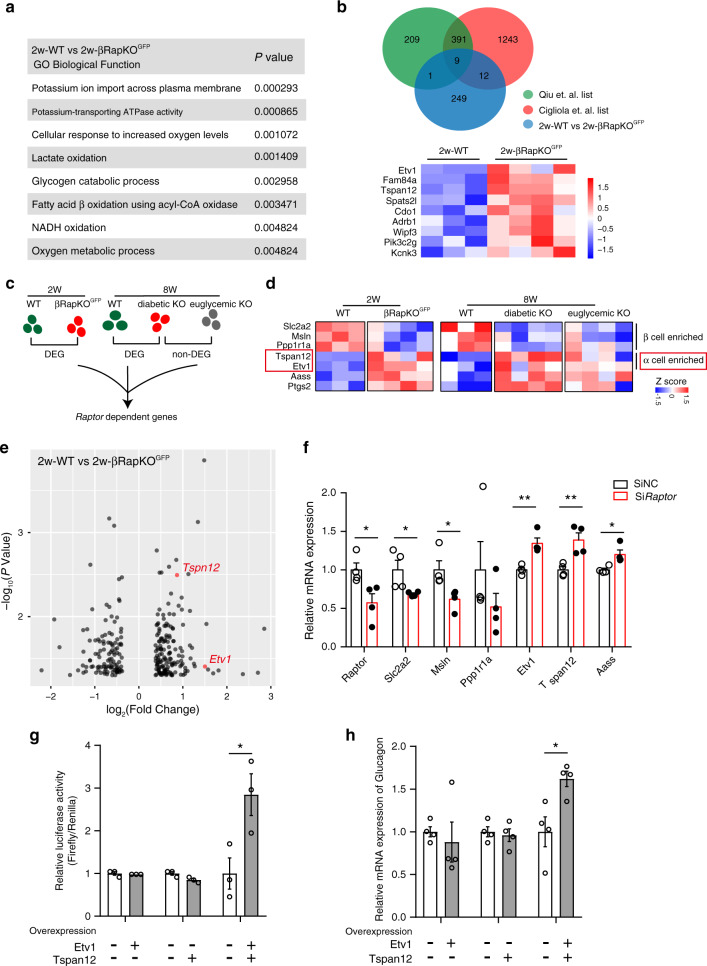
Induction of α-cell-enriched genes in 2-week-old βRapKO^GFP^. **a** GO analysis of differentially expressed genes as identified by microarray of 2-week-old WT (*n* = 3) and βRapKO^GFP^ β-cells (*n* = 4) was associated with β-cell function and metabolism. **b** Venn diagram representation of the subsets of *Raptor*-regulated genes in 2-week-old purified β-cells that were enriched in α-cells and heatmap showed the differential expression of nine overlapped genes in 2-week-old WT and βRapKO^GFP^ β-cells (*n* = 3–4). **c** Analysis strategy to identify *Raptor*-dependent genes in β-cells is shown. **d** Heatmap of seven *Raptor*-dependent genes obtained from 8-week-old RNA-seq and 2-week-old microarray. **e** Volcano plot shows differential genes between 2-week-old WT and βRapKO^GFP^ β-cells. Microarray identification of *Etv1* and *Tspan12* as significantly upregulated α-cell-enriched genes in 2-week-old βRapKO^GFP^ β-cells. **f** INS-1 cells were transfected with Si*Raptor* or SiNC for 48 h. qRT-PCR confirmed *Raptor*-dependent genes in INS-1 cells (*n* = 4 independent cell experiments, for *Raptor*, *p* = 0.029; for *Slc2a2*, *p* = 0.048; for *Msln*, *p* = 0.031; for *Ppp1r1a*, *p* = 0.28; for *Etv1*, *p* = 0.004; for *Tspan12*, *p* = 0.008; for *Aass*, *p* = 0.02). **g**, **h** INS-1 cells were transfected with GFP, Etv1, or Tspan12 overexpression vector. **g** Luciferase reporter assay using a rat glucagon promoter reporter (*n* = 3 independent cell experiments, *p* = 0.039). **h** qRT-PCR analysis of *glucagon* expression in vector transfected INS-1 cells (*n* = 4 independent cell experiments, *p* = 0.02). Data represent means ± SEM. *p < 0.05, **p < 0.01 by two-sided Student’s *t-*test.

In order to identify *Raptor*-dependent genes responsible for identity change, we performed transcriptomic analyses on purified β-cells sorted from 2-week-old euglycemic βRapKO^GFP^ and WT mice. GO analysis revealed that the differentially expressed genes in 2-week-old βRapKO^GFP^ were enriched in potassium ion transport, lactate oxidation, NADH oxidation, and fatty acid β-oxidation (Fig. 6a). Based on the reference lists by Qiu et al.^31^ and Cigliola et al.^32^, in 2-week-old purified GFP sorted β-cells, *Raptor* deficiency upregulated a subset of α-cell-enriched genes (9 genes), i.e. *Etv1*, *Fam84a*, *Tspan12*, *Spats2l*, *Cdo1*, *Adrb1*, *Wipf3*, *Pik3c2g*, and *Kcnk3* (Fig. 6b). We further compared the transcriptome analysis from 2- and 8-week-old datasets, and identified *Raptor*-dependent genes as those are preferentially changed at both 2- and 8-week-old euglycemic βRapKO^GFP^ β-cells (Fig. 6c). This combined analysis further uncovered seven *Raptor*-dependent genes, including three β-cell enriched genes (*Slc2a2*, *Msln*, *Ppp1r1a*) and two α-cell enriched genes (*Etv1*, *Tspan12*) (Fig. 6d,e), possibly involved in β-cell identity and plasticity maintenance. We confirmed the downregulation of *Slc2a2*, *Msln*, and *Ppp1r1a*, and upregulation of *Etv1* and *Tspan12* in *Raptor*-deficient INS-1 cells (Fig. 6f). To determine if *Etv1* and *Tspan12* had direct impact on β to α reprogramming, we overexpressed them in INS-1 cells. Overexpression of *Etv1* or *Tspan12* alone did not result in changes in glucagon promoter activity and *glucagon* mRNA expression. Importantly, co-expression of *Etv1* and *Tspan1*2 caused a significant increase in both glucagon promoter activity and *glucagon* mRNA expression (Fig. 6g, h). Unlike some other α-cell-enriched genes *Corin*, *Fam49a*, and *Cela1*, loss of *Raptor*-induced *Etv1* and *Tspan12* upregulation were not reversed by downregulation of *MafB* (Supplementary Fig. 7), indicating a *MafB*-independent manner. These results suggest that *Raptor* directly suppress α-cell enriched genes, such as *Etv1* and *Tspan12* in healthy β-cells and prevent β-cell default.

## Discussion

There is increasing evidence that loss of β-cell identity and the acquisition of multi-hormonal cells are occurring in the islets of patients with diabetes^38,39^. However, it is unclear whether these events participate in the initiation of disease or secondary to hyperglycemia during disease progression. Moreover, the pathway basis of these reprogramming events has not yet been elucidated. In the present study, we uncovered the essential role of mTORC1 in the maintenance of β-cell identity and the repression of α-cell programs. Loss of *Raptor* resulted in β-cell dedifferentiation: (1) downregulation of β-cell-enriched genes, including key transcriptional factors, glucose metabolism genes, and protein secretory pathway genes; (2) the concomitant upregulation of β-cell disallowed genes; and (3) the likely upregulation of β-cell progenitor markers, i.e. *Ngn3*, *Aldh1a3*, *Sox9*. Moreover, loss of *Raptor* resulted in the appearance of α-cell features in lineage-labeled GFP^+^ β-cells, manifested by gaining the expressions of glucagon and α-cell key transcription factors MafB/Arx, displaying both β- and α-like secretory granules and induction of a group of α-cell enriched genes. Importantly, when 4-week-old *Raptor*-deficient mice were maintained at normoglycemia for 4 weeks, the euglycemic mutant GFP^+^ β-cells still exhibited downregulation of *Nkx6.1*, *Pdx1*, and *Glut2*, upregulation of progenitor markers and a group of α-cell enriched genes, as well as ectopic expression of dedifferentiation marker ALDH1A3. There remained 13-fold increase in Gcg^+^GFP^+^ multi-hormonal cells in euglycemic mutants compared to WT, whereas hyperglycemia indeed further propelled the reprograming process. These data demonstrate that in the absence of mTORC1, loss of β-cell identity and the acquisition of non-β cell program could not be completely reversible upon normalization of glucotoxicity.

Interestingly, in 2-week-old euglycemic βRapKO^GFP^ β-cell, α-cell critical transcription factor *MafB* and a group of α-cell-enriched genes were already strongly induced. We provided evidence that mTORC1 regulated MafB promotor activity and expression via modulating the C/EBPβ isoform ratio of LAP and LIP^37,40^, thus participated in β to α reprogramming. Our data were consistent with a previous observation that ectopic expression of LAP and LIP in monocytes differentially activated MafB expression and osteoclast-genesis^41^. MafB has been shown to activate cell type-specific expression of the glucagon gene^33^. In the present study, we found *Raptor*/C/EBPβ/*MafB* not only regulates *glucagon* mRNA expression but also controls the transcription levels of some α-cell-enriched genes, i.e. *Corin*, *Cela1*, and *Fam49a*. A further combined analysis on 2- and 8-week-old transcriptome revealed seven genes that were *Raptor* specifically dependent, including α-cell enriched genes *Etv1* and *Tspan12*. *Etv1* belongs to the Pea3 group of the ETS family of transcription factors^42–44^ and *Tspan12* is a known component of Tetraspanin (*Tspan12*) family^45–47^; however, their functions in islet β-cell and α-cell are still unknown. Our data showed that co-expression of *Etv1* and *Tspan12* caused a significant increase in glucagon promoter activity and *glucagon* mRNA expression in β-cells. This is relevant since β-cell-specific ETV-dependent genes was significant enrichment of diabetes and obesity-associated genes, thus involving the pathogenesis of diabetes^48^. To our knowledge, this is the first time a metabolic pathway has been identified not only maintaining the proper expression of β-cell functional genes but also repressing non-β cell genes. Conceptually similar findings were reported for *Pdx1* (prevent glucagon)^6^, *Nkx6.1* (prevent somatostatin)^7^, *Nkx2.2* (prevent somatostatin)^9^, and *Dnmt1* (prevent glucagon)^49^ in preventing acquisition of non-β-cell hormones.

Previous studies have illustrated that metabolic defect predisposes to β-cell dedifferentiation and reprogramming^2,50^. Failure β-cells show conjoined features of two cardinal processes: mitochondrial dysfunction and progenitor-line features^5^. *Raptor*-deficient dedifferentiated β-cell was characterized by low UCN3, low GLUT2, and high ALDH1A3 expression, with destabilized β-cell metabolic coupling of insulin secretion. Euglycemic *Raptor*-deficient β-cell exhibited downregulation of mitochondria genes (*Nqo1*, *Atp4a*, *mt-Co3*, *mt-Nd2*, *mt-Co1*, and *Cox6a2)*, decreased *Glut2* expression, derepressed disallowed genes (*HK1* (ref. ^51^), *AldoB*^52^, and *Ldha*^53^) and reduced ATP levels for GSIS. It has been reported that mTORC1 controls mitochondrial dynamics, possibly due to the preferential translational regulation of a subset of cellular mRNA for essential nucleus-encoded mitochondrial proteins^54,55^. The mitochondrial defect in *Raptor*-deficient β-cell was reminiscent of dedifferentiated ALDH^+^ β-cells, in which mitochondrial complex I, IV, and V function were significantly impaired. It is logical that impaired mitochondrial complex I, IV, and V functions can lead to reduced ATP production, stalling of protein translation and reactivation of genes that sustain a cellular progenitor program^56^. We propose that impaired mitochondrial function in *Raptor*-deficient β-cells marks the progression from mitochondria defect to β-cell identity loss and reprogramming in the natural history of β-cell failure before diabetes occurs.

In addition to mitochondria metabolic uncoupling, loss of *Raptor* directly impaired proinsulin synthesis, the most energy consuming processes in β-cell. This was evidenced by dramatic reduction in proinsulin content in euglycemic mutant pancreas and islet, when C-peptide was completely reversed after glucose normalization. On the contrary, the proinsulin/insulin ratio “flaws” detected in diabetic βRapKO^GFP^ by us and Blandino-Rosano et al.^26^ disappeared when mutant mice were constantly kept at normoglycemia. The decreased expression of CPE, a pancreatic convertase, in diabetic βRapKO^GFP^ mice^26^, was reversed in euglycemic βRapKO^GFP^ mice. In diabetes, β-cells undergo marked degranulation of mature secretory granule (SG) in response to hyperglycemia, which stimulates secretion. This leads to decreased pancreatic C-peptide and CPE, which also resides in SG. This is found in βRapKO^GFP^ mice, which are insulin deficient to begin with, as well as in other models of diabetes^57,58^. Treatment with insulin prevents hyperglycemia and restores the SG pool and consequently increases insulin (C-peptide) content, along with reduced proinsulin/C-peptide ratio. Increased proinsulin/C-peptide ratio per se may represent accumulation of young SG, as the mature granules are being secreted; whether it also imply the processing defects needs to be confirmed by pulse-chase experiments. Likewise, genes involved in ROS production and anti-inflammatory process were also recovered after glycemic normalization. This is consistent with the finding that chronic hyperglycemia is highly associated with upregulation of inflammatory mediators in both animals and in human diabetic subjects^59^, while insulin can ameliorate the inflammatory responses^60^. Moreover, hyperglycemia increased ROS production by driving mitochondria toward increased oxygen use and decreased ATP formation^61,62^, which in turn suppressed insulin generation in pancreatic β-cells by inhibiting insulin gene transcription factors such as Pdx1 or MafA^63,64^. Taken together, our work identified the importance of mTORC1 signaling for metabolic coupling and proinsulin synthesis in β-cells, while attributed the inappropriate proinsulin/insulin ratio, ROS, and inflammatory responses in βRapKO^GFP^ to glucotoxicity.

**Fig. 7:**
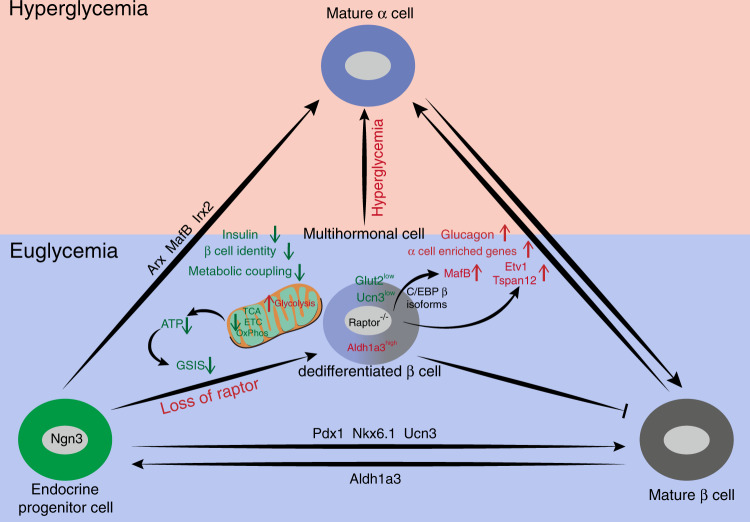
The role of *Raptor* in β-cell identity maintenance and α-cell gene repression. *Raptor* is required for maintaining β-cell identity as well as repressing α-cell signature. At euglycemia, loss of *Raptor* results in β-cell dedifferentiation (marked as UCN3^low^, Glut2^low^, and Aldh1a3^high^) with compromised β-cell identity, metabolic uncoupling, and regression to multi-hormonal state with α-cell features. We propose *Raptor*/C/EBPβ/*MafB*-dependent and *MafB*-independent ways in silencing α-cell-enriched genes and glucagon expression in healthy β-cells. *Raptor*-deficient β-cells with compromised identity become dysfunctional and eventually cause diabetes; in turn, hyperglycemia further aggravates dedifferentiation and reprogramming process.

In light of the current data, we propose *Raptor* controls β-cell anabolic metabolism and serves as checkpoint of β-cell identity and plasticity (Fig. 7). At euglycemia, loss of *Raptor* results in β-cell dedifferentiation (marked as UCN3^low^, GLUT2^low^, and ALDH1A3^high^) with compromised β-cell identity, metabolic uncoupling, and regression to multi-hormonal state with α-cell features. We propose *Raptor*/C/EBPβ/*MafB*-dependent and *MafB*-independent ways in silencing α-cell-enriched genes and glucagon expression in healthy β-cells. *Raptor*-deficient β-cells with compromised identity become dysfunctional and eventually cause diabetes; in turn, hyperglycemia further aggravates dedifferentiation and reprogramming process. Our data provide insights into the molecular nature of dedifferentiation process that independent of metabolic state, and by translating mTORC1-β cell default biology into mechanism-based interventions might help to reverse the course of diabetes.

## Methods

### Mice and supplementation with insulin using osmotic pumps

β-Cell-specific *Raptor* knockout mice (βRapKO) were generated by crossing *Raptor*^flox/flox^ mice (purchased from the Jackson Laboratory, C57BL/6J) with mice expressing Cre recombinase driven by the rat insulin promoter (RIP-Cre, mixed C57BL/6J:129/SvJ) and were further bred to *Rosa26-EGFP* mice (purchased from the Jackson Laboratory, USA) to generate βRapKO^GFP^ mice (*Raptor*^*−/−*^, *RIP-Cre*, *Rosa26*^*GFP*^), permitting lineage tracing of *Raptor*-deleted cells. βRapKO^GFP^ mice were used for experiments and their age-matched littermates (*Raptor*^*+/+*^, *RIP-Cre*, *Rosa26*^*GFP*^) were used as WT controls. Genotyping was performed by PCR using genomic DNA isolated from the toe tips of newborn mice. Only male animals were used for the experiments. Animals were maintained in a specific pathogen-free animal facility with 22 ± 1 °C, 60–70% humidity on a 12-h light–dark cycle. Water and food were provided ad lib. All animal experiments were approved by the Animal Care Committee of Shanghai Jiao Tong University School of Medicine.

Four weeks after birth, some βRapKO^GFP^ mice were anesthetized by intraperitoneal injection of 1% Pelltobarbitalum Natricum and subcutaneously implanted with Alzet mini-osmotic pumps (model 1004; Charles River Laboratories, USA) at a dose of 0.2–0.3 U/day over a period of 28 days supplementing with human insulin (Novolin^®^ R Penfill^®^, Novo Nordisk, Denmark). Blood glucose was measured every other day over the entire period of the experiment. Twenty-eight days after pump implantation, mice were sacrificed and pancreas was removed.

### Metabolic studies

Blood glucose was monitored every 2 weeks. We performed glucose tolerance tests on overnight-fasted mice by intraperitoneal injection of 2 g/kg glucose. Blood glucose concentration was measured before and 15, 30, 60, and 120 min after glucose injection by glucometers (Accu-Chek, Roche, Mannheim, Germany). Six-hour-fasting serum insulin concentration was measured by ELISA Kit (Mouse Ultrasensitive Insulin ELISA Kit, Alpco, USA) and 6 h-fasting serum glucagon concentration was measured by radioimmunoassay kit (Glucagon ELISA Kit, Chemiluminescent, Millipore, USA).

Pancreas was removed and disrupted using ethanol-HCl extraction and homogenizer (Fisher Scientific, USA). Samples were centrifuged, and the supernatant after centrifugation was used for measurement of total C-peptide/proinsulin content per pancreas. The C-peptide and proinsulin content were analyzed by C-peptide (Mouse C-Peptide ELISA Kit, Crystal Chem, USA) and proinsulin (Rat/Mouse Proinsulin ELISA, Mercodia, Sweden) ELISA kits and normalized to pancreas protein concentration (BCA method; ThermoFisher Scientific, USA).

### α-Cell mass and β-cell mass measurement

Whole pancreas from 8-week-old mice were weighed, fixed, embedded, and sectioned continuously. At least 10 evenly 200 μm apart sections throughout the entire pancreas were picked to immunostaining for insulin, glucagon, followed by peroxidase conjugated secondary antibody, visualized using a DAB Peroxidase Substrate Kit (Fuzhou Maixin Biotech, China), and counterstained with eosin^65^. Digital images of whole pancreas were captured by a Nikon MZ 100 microscope (Japan). Total pancreatic and insulin/glucagon positive areas of each section were measured and calculated using Meta-Morph version6.1 (Molecular Devices, USA). β-Cell mass/α-cell mass was obtained by multiplying the ratio of total insulin-positive area (total glucagon positive area) to total pancreatic area with the pancreas weight.

### Immunofluorescence

Pancreas tissues were fixed in 4% paraformaldehyde overnight at 4 °C, embedded in paraffin, and cut into 6-μm-thick section. For nuclear transcription factor detection, we performed antigen retrieval in citrate buffer (Vector Laboratories, USA) for 15 min in the microwave. Slides were washed with PBS and blocked with normal donkey serum (5%), and then primary antibodies were applied (Supplementary Table 1).The Arx antibody was from Dr.Kunio Kitamura’s lab^66^. For specific antibodies, biotin-streptavidin system (Fuzhou Maixin Biotech, China) and Tyramide signal amplification (PerkinElmer, USA) were performed. Fluorescent secondary antibodies were from Invitrogen (USA) (Supplementary Table 2). Slides were mounted with Vectashield with DAPI (SouthernBiotech, USA). We used confocal microscopy (Zeiss710, Germany) and Laser Scanning Microscope Software (Zen (blue edition)/2.1) to survey co-localization and capture images. Immunofluorescent images were analyzed by ImageJ software (edition/1.50i, NIH).

Isolated islets were dispersed into single cells with 0.25% trypsin-EDTA (Gibco, USA), then cells were cultured on a microscope slide (Millipore’s Millicell EZ SLIDE, USA) overnight, washed with PBS and fixed in 4% pre-cooling paraformaldehyde for 20 min, and then the fixed cells were stained for PS6 and insulin.

### Isolation of pancreatic islets and purification of β-cells

Pancreatic islets were isolated by collagenase P (Roche, Switzerland) injecting into the common bile duct. The perfused pancreas was dissected and incubated at 37 °C for 17 min. Digested exocrine cells and intact islets were separated via centrifugation, and intact islets were manually handpicked.

Islets were digested into single cells by 0.25% trypsin-EDTA (Gibco, USA) solution and sorted for GFP by FACS (MoFlo XDP, Beckman Coulter, USA). Cells from *Raptor*^*+/+*^ *Rosa26*^*GFP*^ mice were used as a negative control for FACS gating. β-Cell-derived endocrine cells lineage traced with GFP were purified by FACS sorting to a purity of 85–95%. Sorted cells were then processed for RNA extraction.

### RNA-sequencing analysis

Total RNA was isolated using RNeasy Micro kit (Qiagen, Germany). Paired-end libraries were synthesized by using the TruSeq® RNA Sample Preparation Kit (Illumina, USA) following TruSeq® RNA Sample Preparation Guide. Briefly, the poly-A-containing mRNA molecules were purified using poly-T oligo-attached magnetic beads. Following purification, the mRNA is fragmented into small pieces using divalent cations under 94 °C for 8 min. The cleaved RNA fragments are copied into first-strand cDNA using reverse transcriptase and random primers. This is followed by second-strand cDNA synthesis using DNA Polymerase I and RNase H. These cDNA fragments then go through an end repair process, the addition of a single “A” base, and then ligation of the adapters. The products are then purified and enriched with PCR to create the final cDNA library. Purified libraries were quantified by Qubit® 2.0 Fluorometer (Life Technologies, USA) and validated by an Agilent 2100 bioanalyzer (Agilent Technologies, USA) to confirm the insert size and calculate the mole concentration. Cluster was generated by cBot with the library diluted to 10 pM and then were sequenced on the Illumina HiSeq 2500 (Illumina, USA). The library construction and sequencing was performed at Shanghai Biotechnology Corporation. Sequencing raw reads were preprocessed by filtering out rRNA reads, sequencing adapters, short-fragment reads, and other low-quality reads. We used Tophat v2.1.0 (ref. ^67^) to map the cleaned reads to the mouse mm10 reference genome with two mismatches. After genome mapping, Cufflinks v2.1.1 (ref. ^68^) was run with a reference annotation to generate FPKM values for known gene models. Differentially expressed genes were identified using Cuffdiff^68^. The *p* value significance threshold in multiple tests was set by the false discovery rate (FDR). The fold-changes were also estimated according to the FPKM in each sample. The differentially expressed genes were selected using the following filter criteria: FDR ≤ 0.05 and fold change ≥ 1.5.

### Microarray analysis

Total RNA was extracted from freshly purified β-cells of 2-week-old βRapKO^GFP^ and WT mice using RNeasy Micro Kit (Qiagen, Germany) and sample qualities were assessed using an Agilent Bioanalyzer 2100 (Agilent technologies, Santa Clara, CA, USA), taking a minimal cut-off RIN ≥ 7. We included at least 3–4 independent biological replicates, each composed of RNA isolated from β-cells of 5–6 animals with the same genotype. Total RNA samples of each group were then used to generate biotinylated cRNA targets for the Affymetrix Gene Clariom S Array. Data were analyzed using Genespring software (Agilent).

### qRT-PCR analysis and western blotting

For qRT-PCR, total RNA was extracted by TRIzol reagent (Invitrogen, USA) according to the manufacturer’s protocols. One microgram of RNA was used for synthesis of cDNA using SuperScriptII (Invitrogen, USA) with random primer as described. Duplicate samples for quantitative PCR were run in an ICycler (ABI, California, USA, QuantStudio 12K Flex Software/v1.2.2). ΔCt for each gene was determined after normalization to actin, and ΔΔCt was calculated relative to the control. Gene expression values were then expressed as a fold change, calculated by 2^−ΔΔCt^. Primer sequences can be obtained in Supplementary Table 3.

The protein concentration of islet and cell lysates was quantified by BCA protein assay (Pierce, Rockford, IL, USA). Protein samples (20 μg) were separated by electrophoresis on TGX precast gel 4–20% (Bio-Rad, Hercules, CA, USA) and transferred to a polyvinylidene fluoride (PVDF) membrane. Primary antibodies are listed as follows: rabbit anti-RAPTOR (1:1000; Cell Signaling Technology, USA), rabbit anti-PS6 (Ser240/244) (1:1000; Cell Signaling Technology, USA), rabbit anti-4E-BP1 (1:1000; Cell Signaling Technology, USA), rabbit anti-tubulin (1:1000; Cell Signaling Technology, USA), rabbit anti-CPE (1:1000; GeneTex, USA), and guinea pig polyclonal anti-insulin antibody from Liuming Lab^69^. TUBULIN was used as an internal control to normalized band intensity. Horseradish peroxidase-coupled goat-anti-rabbit IgG (1:2000; Cell Signaling Technology, USA) and horseradish peroxidase-coupled goat-anti-guinea pig IgG (1:2000; Jackson ImmunoResearch, USA) were used as a secondary antibody. Blots were developed with enhanced chemiluminescence (Millipore, USA) and detection was performed using LAS-4000 (GE, USA) and Image Studio Software version 5.2 (LI-COR).

### In vitro GSIS and proinsulin/C-peptide measurement

Isolated islets were recovered in 1640 RPMI supplemented with 10% serum at a 11.1 mM glucose. Then the islets were pre-incubated for 1 h at 37 °C in Krebs–Ringer bicarbonate HEPES buffer (KRBH) supplemented with 0.2% BSA in the presence of 2.8 mM glucose, followed by 1 h of incubation in KRBH supplemented with 0.2% BSA in the presence of 2.8 mM glucose or 16.7 mM glucose. The supernatant was collected and C-peptide content was extracted by acid ethanol. C-peptide in supernatant and islet lysates were measured by C-peptide ELISA assay (Mouse C-Peptide ELISA Kit, Crystal Chem, USA). Secreted C-peptide was calculated as the percentage of total C-peptide content per hour. Islet proinsulin and C-peptide content were measured using the islet lysates by Proinsulin (Rat/Mouse Proinsulin ELISA, Mercodia, Sweden) and C-peptide (Mouse C-Peptide ELISA Kit, Crystal Chem, USA) ELISA kits.

ATP levels were measured using the Bioluminescence assay kit (FLASC, Sigma, USA). For ATP measurement, batches of 30 islets each condition following the procedure of GSIS were lysed using the kit buffer at 25 °C for 5 min. ATP was expressed as relative light units using a luminometer (Promega, Madison, WI).

### INS-1 cell culture and luciferase assays

INS-1 cells purchased from the CAMS Cell Culture Center (Beijing, China) were grown in RPMI 1640 medium containing 11.1 mM glucose and supplemented with 10 mM HEPES, 10% FBS, 2mM l-glutamine, 1 mM sodium pyruvate, 5 μM β-mercaptoethanol, 100 IU/mL penicillin and 100 μg/mL streptomycin at 37 °C in a humidified 5% CO_2_ atmosphere. The cells were sub-cultured when they reached 80% confluence. PcDNA3.1-*LAP* plasmid, pcDNA3.1-*LIP* plasmid, pcDNA3.1-*Etv1* plasmid, and pcDNA3.1-*Tspan12* plasmid transfections were conducted using Lipofectamine 2000 (Invitrogen, USA) according to the manufacturer’s instructions.

SiRNA oligonucleotides for *Raptor*, *MafB*, and their control were designed and synthesized from GenePharma (Shanghai, China). Lipofectamine 2000 transfection reagent (Invitrogen, USA) was used according to the manufacturer’s instructions.

INS-1 cells transfected with LAP/LIP, Etv1/Tspan12 plasmid, or GFP plasmid were plated in 24-well plates 24 h before transfection. When the cells reached 70–80% confluence, they were transfected with plasmids (examined promoter plasmid and pRL-SV40) using Lipofectamine 2000 transfection reagent (Invitrogen, USA). Six hours after transfection, the medium was changed. MafB promoter activity was examination after 12 h, and Glucagon promoter activity was determined 24 h later. The cells were lysed in 1× PLB, and luciferase assays were performed using the Dual-Luciferase Reporter Assay System (Promega, USA) as recommended by the manufacturer. All luciferase assay experiments were performed in triplicate.

### Data analysis

Data are presented as means ± SEM and analyzed by two-tailed Student’s *t-*test for two groups and ANOVA analysis for multiple groups. Statistics were computed by SPSS 22.0 (USA). *P* value less than 0.05 was considered to be statistically significant.

### Reporting summary

Further information on research design is available in the Nature Research Reporting Summary linked to this article.

## Supplementary information

Supplementary Information

Reporting Summary

## Data Availability

All data supporting the findings of this study are available with the article. Data from RNA-seq are available under GEO Series GSE130792. Microarray data has been deposited in the NCBI GEO repository with an accession ID GSE140224. Source data for Figs. 1–6 and Supplementary Figs. 1–4, Supplementary Figs. 6 and 7 are provided with the paper as a Source Data File.

## Source Data

Source Data
