# Supplementary material for: Raptor determines β-cell identity and plasticity independent of hyperglycemia in mice
Source: Nat Commun. 2020 May 21;11:2538. doi: 10.1038/s41467-020-15935-0 (PMC7242325; doi:10.1038/s41467-020-15935-0)
Supplement: Supplementary file 1 — Supplementary Information [file 41467_2020_15935_MOESM1_ESM.pdf]

Supplementary Information

Raptor determines β-cell identity and plasticity independent of hyperglycemia in mice

Yin et al

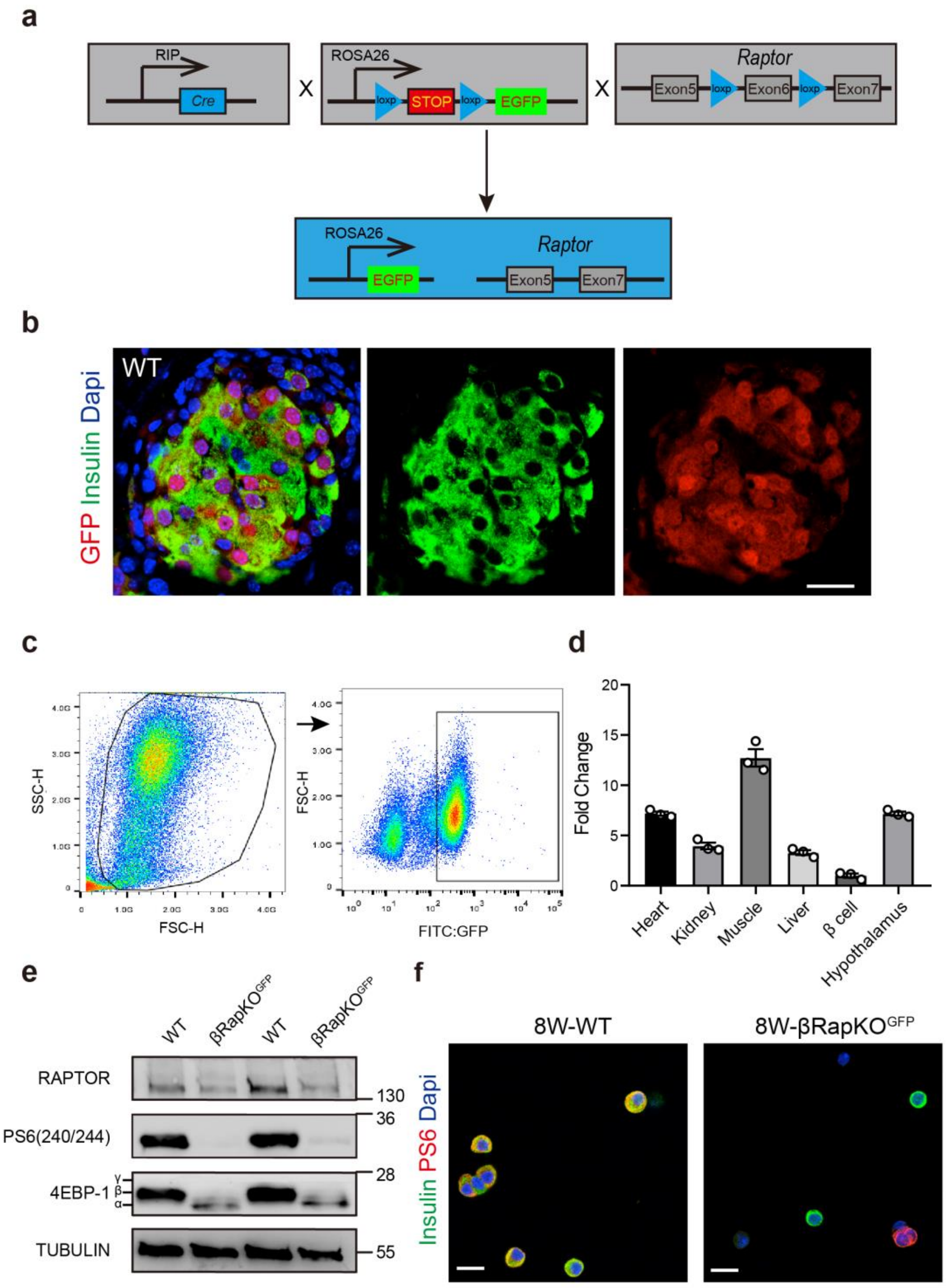

Supplementary Figure 1. Validation of βRapKO^{GFP} mice.

(a) Generation of βRapKO^{GFP} mice. (b) Representative images of the lineage-tracing immunofluorescence-based analysis of 8-week-old islets from *RipCre-Rosa26^{GFP}* mice, insulin (green), GFP (red) (n=3). Scale bars, 20μm. (c) The gating strategy for FACS of GFP^{+} β cells is indicated. (d) Relative expression of *Raptor* in heart, kidney, muscle, liver, β-cell and hypothalamus from 8-week-old βRapKO^{GFP} mice (n=3, means ± SEM). (e) Western blotting showed decreased mTORC1 downstream targets phosphorylation of PS6 (Ser240/244) and 4E-BP1 in 8-week-old WT and βRapKO^{GFP} islets (n=2). (f) Representative images of immunofluorescent staining for PS6 (Ser240/244) (red) and insulin (green) in dispersed islets from 8-week-old control and βRapKO^{GFP} mice (n=3). Scale bars, 10μm.

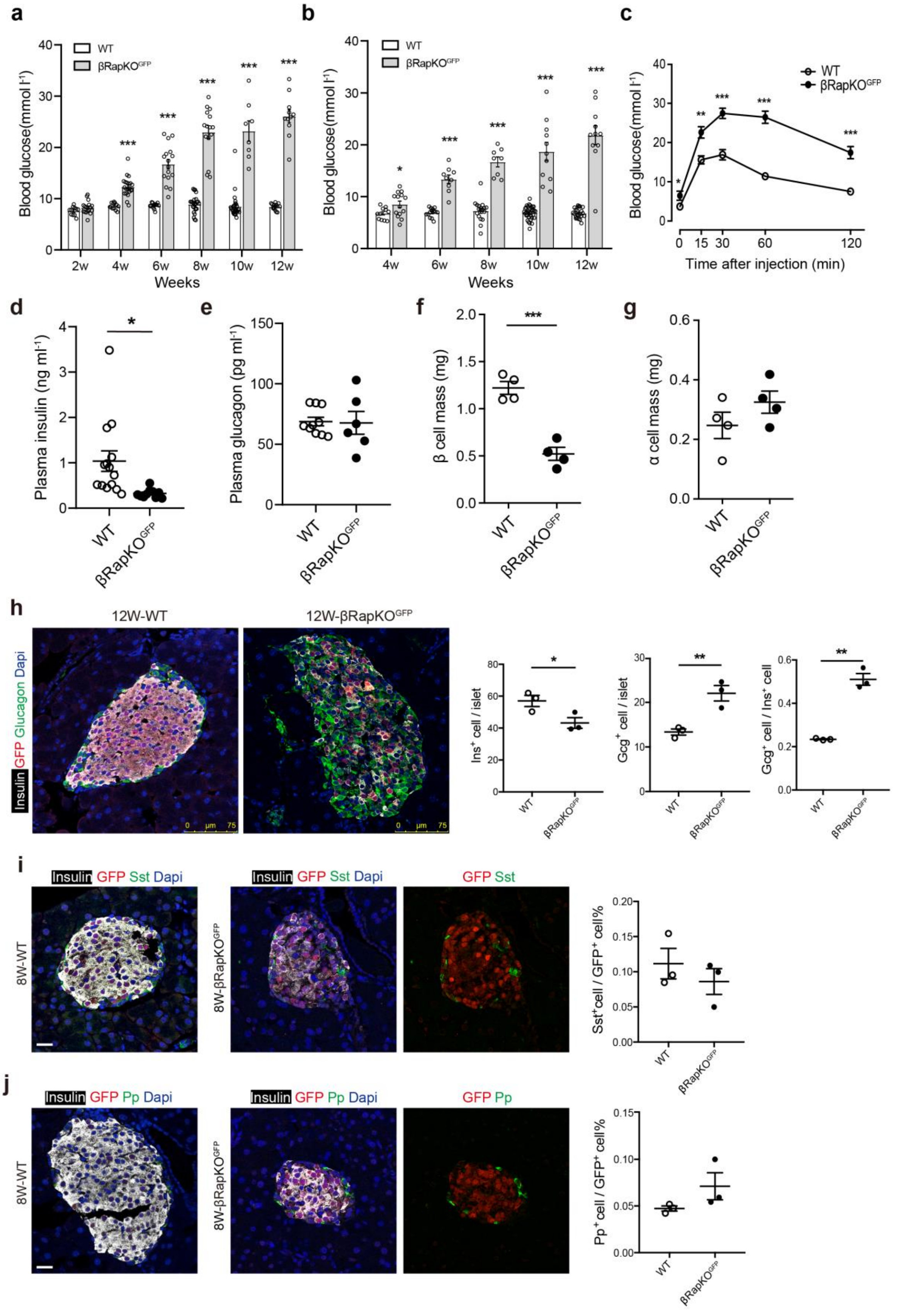

Supplementary Figure 2. Impaired glucose tolerance and changed islet structure in βRapKO^{GFP} mice. (a) Random blood glucose (n= at least 11 for WT, n= at least 8 for βRapKO^{GFP}, p values included in source data), and (b) 6-h fasting blood glucose were monitored every 2 weeks in WT and βRapKO^{GFP} mice (n= at least 12 for WT, n= at least 8 for βRapKO^{GFP}, p values included in source data). (c) Intraperitoneal glucose tolerance tests were performed on 8-week-old WT and βRapKO^{GFP} mice (n=7 for WT, n=6 for βRapKO^{GFP},

p values included in source data). (d) 6-h fasting plasma insulin concentrations (n=14 for WT, n=10 for βRapKO^{GFP}, p=0.015) and (e) 6-h fasting plasma glucagon concentrations in 8-week-old WT and βRapKO^{GFP} mice were shown (n=10 for WT, n=6 for βRapKO^{GFP}, p=0.90). (f) Pancreatic β-cell mass (n=4 independent samples for each group, p=0.00035) and (g) α-cell mass were shown (n=4 independent samples for each group). (h) Representative images of pancreatic sections from 12-week-old WT and βRapKO^{GFP} mice were immunostained for insulin (white), glucagon (green) and GFP (red) (n=3). The number of Ins$^+$ cells per islet (p=0.047), the number of Gcg$^+$ cells per islet (p=0.009) and quantification of Gcg$^+$/Ins$^+$ cell ratio (p=0.009) were calculated in 12-week-old WT and βRapKO^{GFP} mice (n=3). At least 46 islets were used for quantifications. Scale bars, 75μm. (i) Representative images of pancreatic sections from 8-week-old WT, βRapKO^{GFP} mice were immunostained for insulin (white), somatostatin (green) and GFP (red). The percentage of GFP$^+$Sst$^+$ cells among GFP$^+$ cells in 8-week-old WT and βRapKO^{GFP} mice (n=3). At least 43 islets were used for quantifications. Scale bars, 20μm. (j) Representative images of pancreatic sections immunostained for insulin (white), pancreatic polypeptide (green) and GFP (red) in 8-week-old WT and βRapKO^{GFP} mice. The percentage of GFP$^+$Pp$^+$ cells among GFP$^+$ cells in 8-week-old WT and βRapKO^{GFP} mice (n=3). At least 43 islets were used for quantifications. Scale bars, 20μm. Data represent means ± SEM. *p < 0.05, **p < 0.01, ***p< 0.001 by two-sided Student's t test.

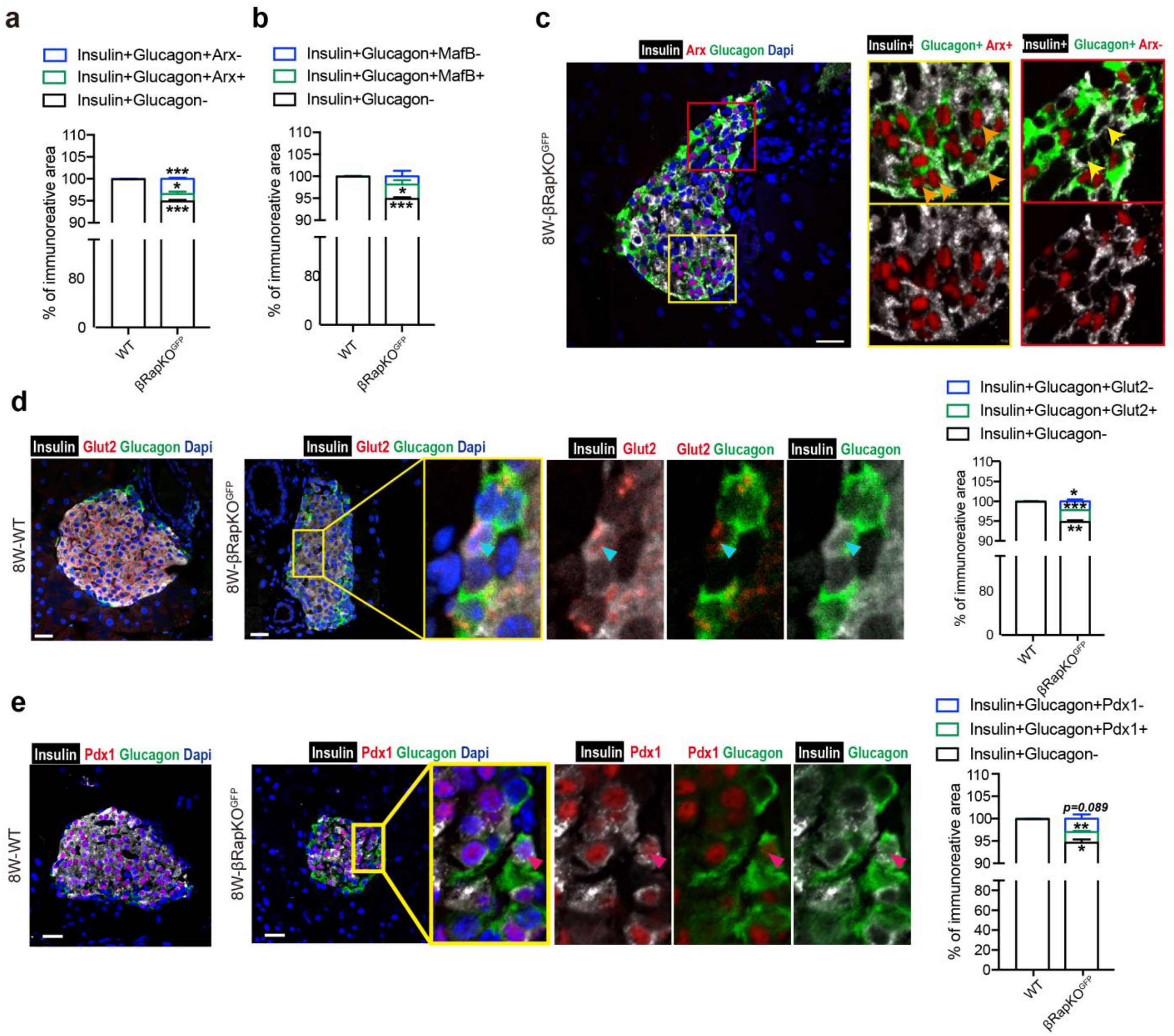

Supplementary Figure 3. Adult β-cells acquire islet α-like features in βRapKO^{GFP} mice.

(a-b) Percentage of Arx$^+$Insulin$^+$Glucagon$^+$ (for tri-positive cells, p=0.037, for double-positive cells, p=0.000022, for only insulin positive cell, p=0.00002) (a) and MafB$^+$Insulin$^+$Glucagon$^+$ (for tri-positive cells, p=0.029, for double-positive cells, p=0.12, for only insulin positive cell, p=0.00002) (b) cells were calculated (n=3). At least 50 islets or 2000 insulin positive cells were used for quantifications. (c) Representative images of Arx (red) positive and negative subpopulations in insulin (white) and glucagon (green) double positive cells (n=3). Boxes showed the specific area of the islet, which were enlarged: orange arrows for Arx$^+$ cells, yellow arrows for Arx$^-$ cells. (d) Representative images of immunofluorescence labeled with insulin (white), Glut2 (red) and glucagon (green) in 8-week-old WT and βRapKO^{GFP} mice (n=3). Percentage of Glut2$^+$Insulin$^+$Glucagon$^+$ was calculated (n=3, for tri-positive cells, p=0.0000004, for double-positive cells, p=0.014, for only insulin positive cell, p=0.002). At least 50 islets or 2000 insulin positive cells were used for quantifications. (e) Co-immunostaining of Pdx1 (red), insulin (white) and glucagon (green) in 8-week-old βRapKO^{GFP} mice compared with WT mice (n=3). Percentage of Pdx1$^+$Insulin$^+$Glucagon$^+$ was calculated (n=3, for tri-positive cells, p=0.001, for double-positive cells,

p=0.089, for only insulin positive cell, p=0.018). At least 50 islets or 2000 insulin positive cells were used for quantifications. Yellow boxes showed the specific areas of the islet, which were enlarged and represented by arrows on the right to demonstrate protein expression within specific cells. Scale bars, 20μm. Data represent means ± SEM. *$p < 0.05$, **$p < 0.01$, ***$p < 0.001$ by two-sided Student's t test.

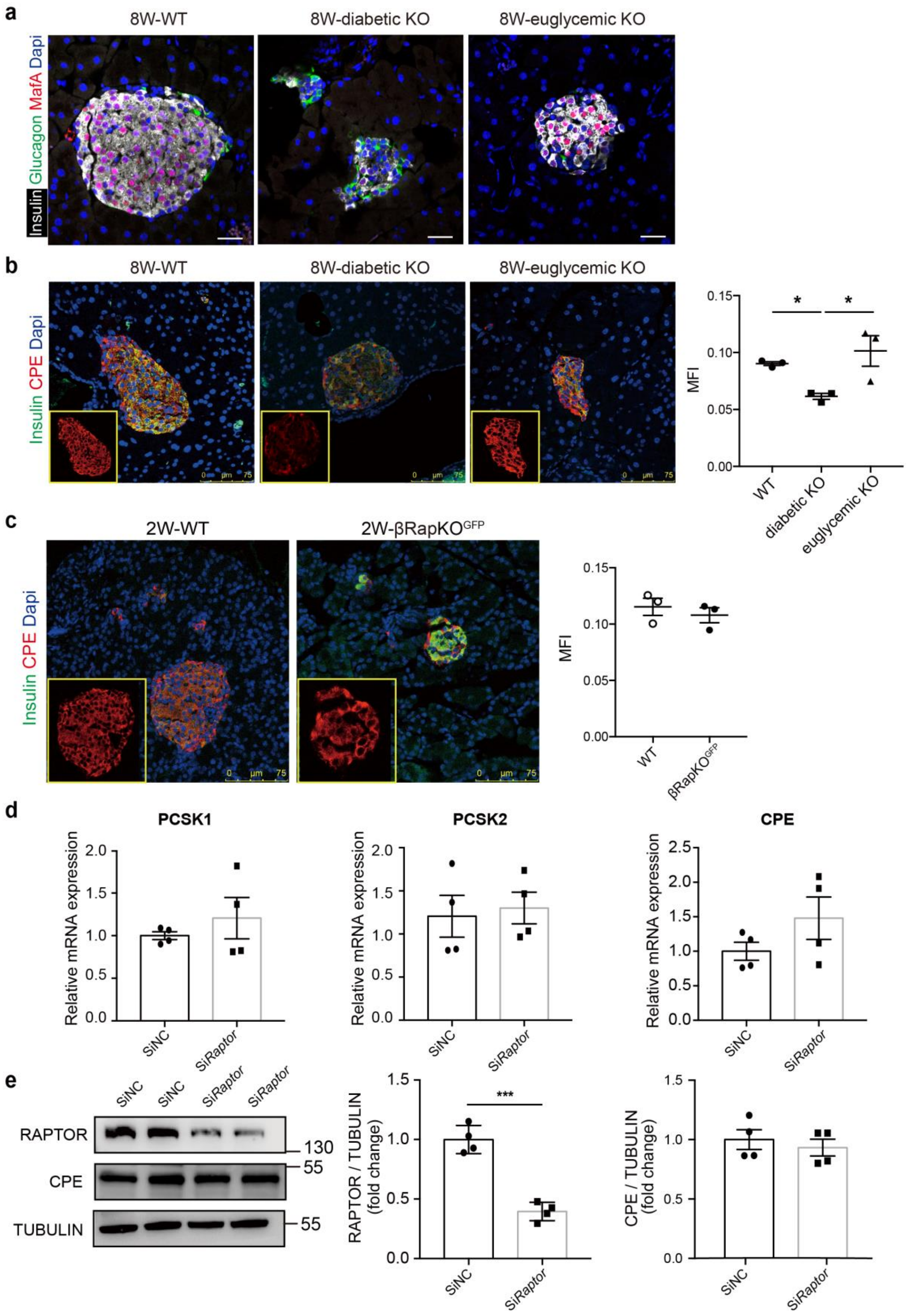

Supplementary Figure 4. mTORC1 does not directly influence CPE expression.

(a) Representative images of immunofluorescence staining for insulin (white), glucagon (green) and MafA (red) in 8-week-old WT, diabetic βRapKO^{GFP} and euglycemic βRapKO^{GFP} islets (n=3). Scale bars, 20μm. (b) Representative immunofluorescent staining for CPE (red) and insulin (green) among 8-week-old WT, diabetic

βRapKO^{GFP} and euglycemic βRapKO^{GFP} mice. Insets showed different expression levels of CPE. MFI of CPE in WT, diabetic βRapKO^{GFP} and euglycemic βRapKO^{GFP} islets (n=3, one way ANOVA, LSD multiple comparison, WT vs diabetic βRapKO^{GFP}: p=0.044; diabetic βRapKO^{GFP} vs euglycemic βRapKO^{GFP}: p=0.012; WT vs euglycemic βRapKO^{GFP}: p=0.361). At least 10 islets from 3 sections were used for quantifications of MFI. Scale bars, 75μm. (c) Representative immunofluorescent staining for CPE (red) and insulin (green) in 2-week-old WT and βRapKO^{GFP} mice (n=3). Insets showed expression levels of CPE. MFI of CPE in β cells. At least 10 islets from 3 sections were used for quantifications of MFI. Scale bars, 75μm. (d-e) INS-1 cells were transfected with Si*Raptor* or SiNC for 72h. (d) Relative expression of *Pcsk1*, *Pcsk2* and *CPE* in INS-1 cells transfected with SiNC or Si*Raptor* by qRT-PCR (n=4 independent cell experiments). (e) Representative pictures of Western blotting for RAPTOR and CPE in INS-1 cells transfected with SiNC or Si*Raptor* were shown. Band intensities of RAPTOR and CPE normalized for the corresponding TUBULIN intensity were calculated (n=4 independent cell experiments, p=0.00014). Data represent means ± SEM. *p < 0.05, ***p< 0.001 by two-sided Student's t test and one-way ANOVA.

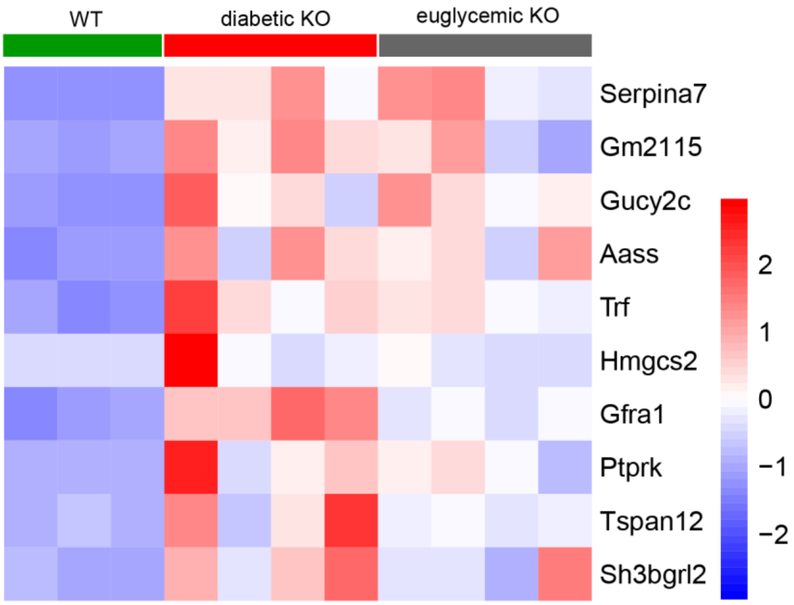

Supplementary Figure 5. Heatmap of other disallowed genes upregulated after *Raptor* Knock-out. Heatmap of other disallowed genes upregulated after *Raptor* knock-out in WT (n=3), diabetic βRapKO^{GFP} (n=4) and euglycemic βRapKO^{GFP} (n=4) β cells.

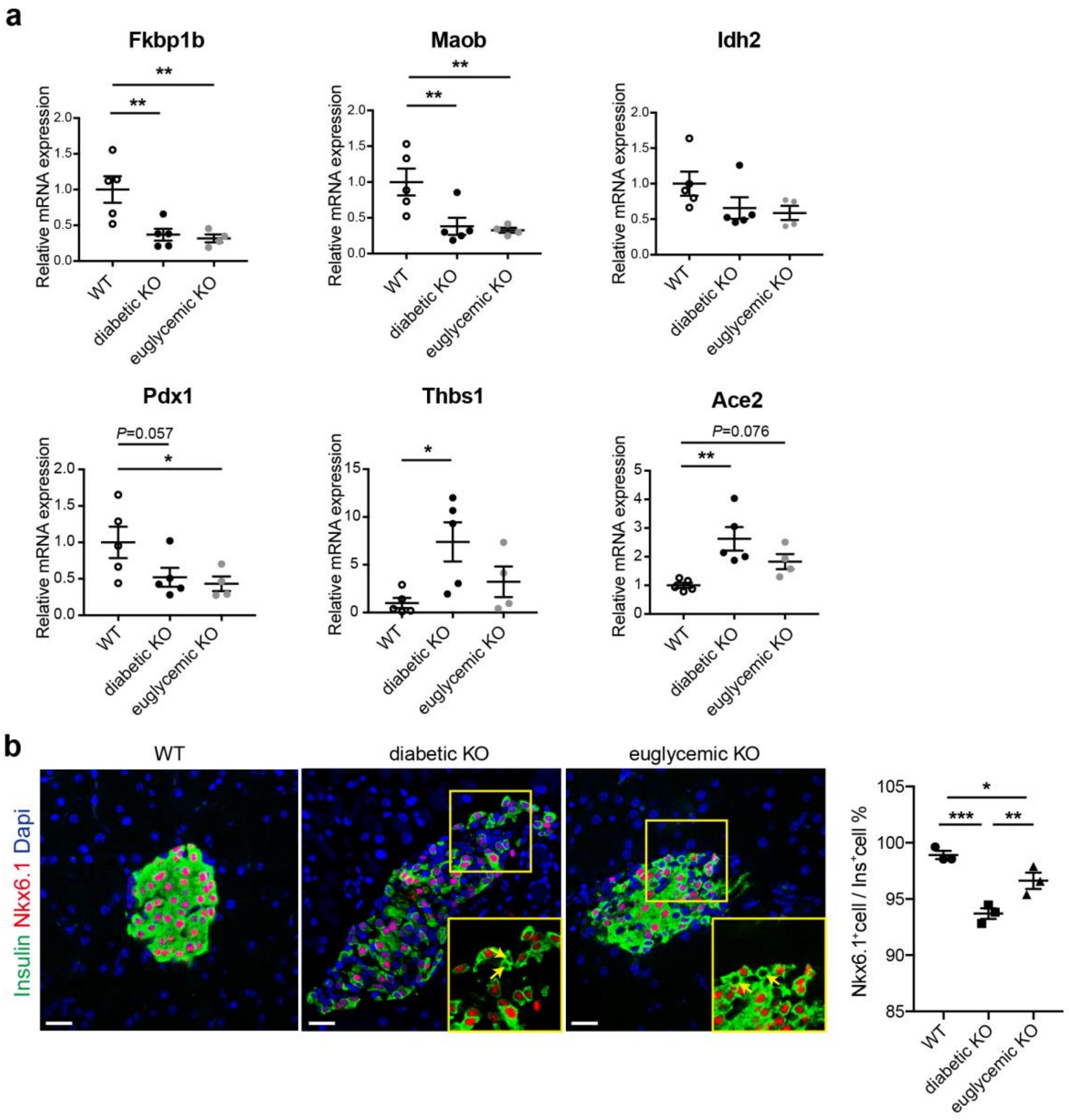

Supplementary Figure 6. β-cell identity and function is not fully corrected in euglycemic βRapKO^{GFP} mice.

(a) Relative expression of genes involved in β-cell function and cell identity by qRT-PCR (n=5 independent samples for WT, n=5 independent samples for diabetic βRapKO^{GFP}, n=4 independent samples for euglycemic βRapKO^{GFP}, p values included in source data). (b) Representative images of pancreatic sections co-immunostaining for insulin (green) and Nkx6.1 (red) (n=3). Percentage of Nkx6.1^{+}Ins^{+} cells among Ins^{+} cells in WT, diabetic βRapKO^{GFP} and euglycemic βRapKO^{GFP} mice was calculated (n=3, WT vs diabetic βRapKO^{GFP}: p<0.001; diabetic βRapKO^{GFP} vs euglycemic βRapKO^{GFP}: p=0.009; WT vs euglycemic βRapKO^{GFP}: p=0.024). At least, 31 islets were used for quantifications. Scale bars, 20µm. Data represent means ± SEM. *p < 0.05, **p < 0.01, ***p< 0.001 by one-way ANOVA.

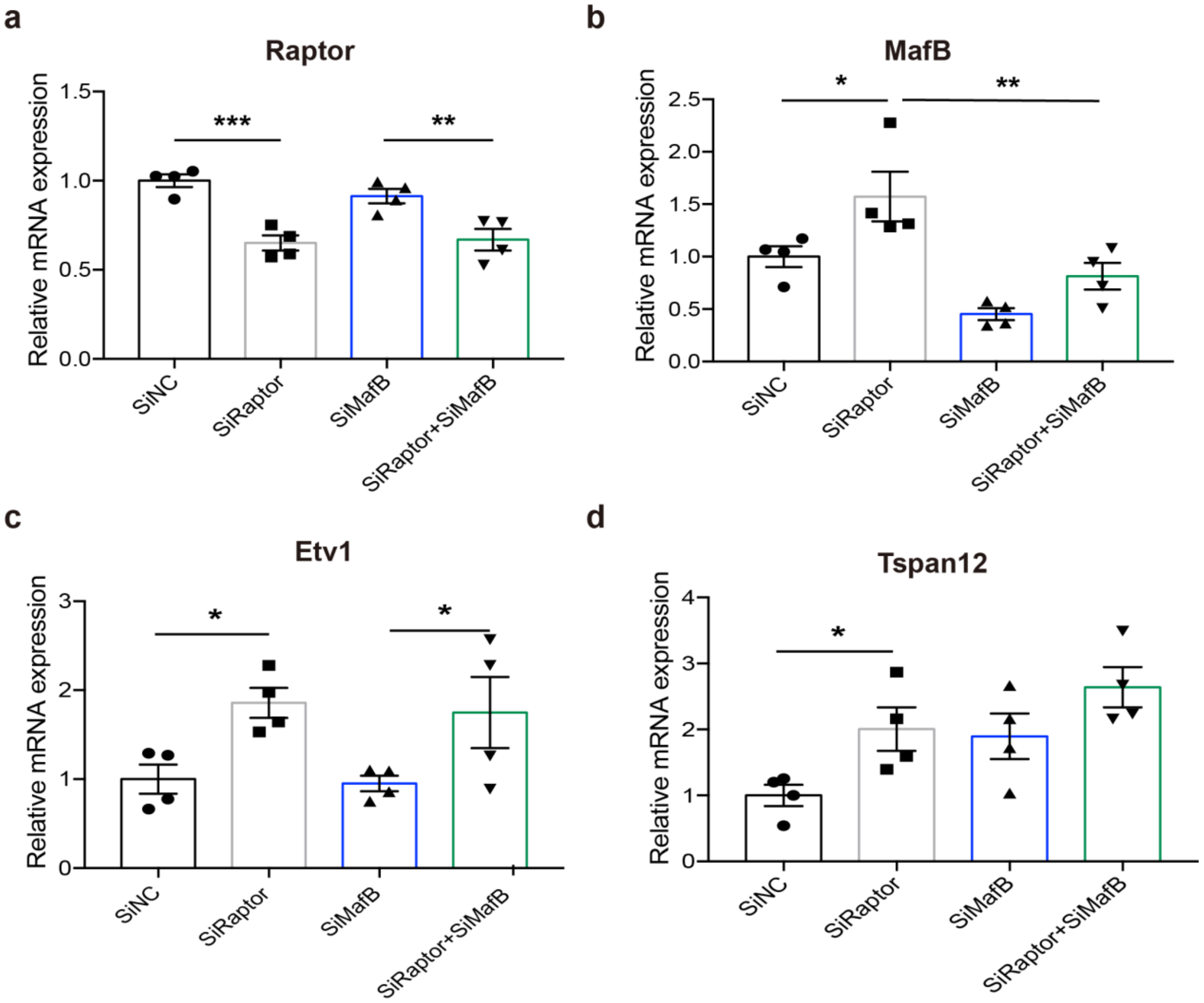

Supplementary Figure 7. Etv1 and Tspan12 are not regulated by MafB.

(a-d) Relative expression of *Raptor* (n=4 independent cell experiments, SiNC vs Si*Raptor*, p<0.001; Si*MafB* vs Si*Raptor*+Si*MafB*, p=0.003) (a), *MafB* (n=4 independent cell experiments, SiNC vs Si*Raptor*, p=0.017; Si*Raptor* vs Si*Raptor*+Si*MafB*, p=0.003) (b), *Etv1* (n=4 independent cell experiments, SiNC vs Si*Raptor*, p=0.025; Si*MafB* vs Si*Raptor*+Si*MafB*, p=0.034) (c) and *Tspan12* (n=4 independent cell experiments, SiNC vs Si*Raptor*, p=0.033) (d) in INS-1 cells which were transfected with SiNC or Si*Raptor* in the presence or absence of Si*MafB* by qRT-PCR. Data represent means ± SEM. *p < 0.05, **p < 0.01, ***p< 0.001 by one-way ANOVA.

Supplementary Table 1. Primary antibodies used in immunofluorescence.

Primary Antibody	Source	Dilution	Antigen Retrieval	Amplification
Guinea pig anti-Insulin	Dako A0564	1:800	No	No
Mouse anti-Glucagon	Abcam K79bB10	1:800	No	No
Rabbit anti-Arx	A kind gift from Dr Kunio Kitamura	1:1000	Yes	No
Mouse anti-Nkx6.1	DSHB F55A10	1:1000	Yes	Yes
Rabbit anti-Pdx1	Abcam ab47267	1:1000	Yes	No
Rabbit anti-Somatostatin	Millipore MAB354	1:400	No	No
Rabbit anti-MafA	Bethyl laboratories IHC-00352	1:1000	Yes	Yes
Rabbit anti-Glut2	Millipore 400061	1:400	Yes	No
Rabbit anti-Pancreatic Polypeptide	Millipore AB939	1:400	No	No
Goat anti-GFP	Rockland	1:400	Yes	No
Rabbit anti-insulin	Cell Signaling Technology 3014	1:500	No	No
Rabbit anti-Aldh1a3	Novus NBP2-15339	1:400	Yes	No
Rabbit anti-Ucn3	Sigma HPA038281	1:400	Yes	No
Rabbit anti-Ki67	Bethyl laboratories IHC-00375	1:500	Yes	No
Rabbit anti-MafB	Bethyl laboratories IHC-00351	1:100	Yes	No
Rabbit anti-CPE	GeneTex GTX33060	1:1000	Yes	No

Supplementary Table 2. Secondary antibodies used in immunofluorescence.

Secondary Antibody	Wavelength	Dilution	Catalog#
Donkey anti-Mouse	Alexa Fluor 594	1:500	A-21203
Donkey anti-Rabbit	Alexa Fluor 594	1:500	A-21207
Donkey anti-Rabbit	Alexa Fluor 488	1:500	A-21206
Goat anti-Guinea Pig	Alexa Fluor 488	1:500	A-11073
Goat anti-Guinea Pig	Alexa Fluor 647	1:500	A-21450
Donkey anti-Goat	Alexa Fluor 594	1:500	A-11058

Supplementary Table 3. Primers sequences used for qRT-PCR in the study.

Primer Sequence	Forward	Reverse
Primers for Mouse		
Glut2	TCTTCACGGCTGTCTCTGTG	AATCATCCCGGTTAGGAACA
Aldh1a3	AGGCTGTATTAAGACCTTCAG	GGAAGTTCCATGGTGTAATG
Cox6a2	TACCCTCACCTGCGCATCAG	GATTGTGAAAAGCGTGTGGTTG
Dlk1	CCCAGGTGAGCTTCGAGT	GGAGAGGGGTACTCTTGTTG AG
Ldha	GGAGAGGGGTACTCTTGTT GAG	TCAGGAGTCAGTGTCACCTTC ACA
AldoB	AGAAGGACAGCCAGGGAAAT	GTTCAGAGAGGCCATCAAGC
Hk1	GTGGACGGGACGCTCTAC	TTCACTGTTTGGTGCATGATT
Slc30a8	CAGAGAACTTCGACAGAAG CC	CTTGCTTGCTCGACCTG TT
Sytl4	ATCATTTAGTGTGCCGAGAA TGC	CCTGTTCGGTAATCAAAG CGA
Rab37	GTCTGCTTGGCTACCTCTGG	GAACCCAGGTGGAAAGTTGA
Glucagon	CATTCACCAGCGACTACAGCAA	TCATCAACCACTGCACAAAATCT
Arx	TTCCAGAAGACGCACTACCC	TCTGTCAGGTCCAGCCTCAT
MafB	AGGACCTGTACTGGATGGC	CACTACGGAAGCCGTCGAAG
Irx2	ACGCACACCACCGGAATG	ATGGATAGGCCGCACTGC
Pyy	AGCGGTATGGGAAAAGAGAA GTC	ACCACTGGTCCACACCTT CTG
Ppy	CCCCTGGAGCCAATGTACC	GAGCAGGGAATCAAGCCAAC
Slc38a5	CCTGCCCACCACCCGTAACC	TGACTCCAGTGTGTGCCATGGC
Cadm1	ATCCCCACAGGTGATGGACA GAATCT	ATTATAGCTGTGTCTGCGTCT GCTGC
Raptor	TTTGTCTACGACTGTTCCAA TGC	GCTACCTCTAGTTCCTGCTCC
Thbs1	ACTTCACCTTTGCCACCTC	AGACTCTGGAATGCGGTTG

Nqo1	GGTTTACAGCATTGGCCACACT	AACAGGCTGCTTGGAGCAAA
Fkbp1b	ATGGGCGTGGAGATCGAGAC	GTAGCTCCATAGGCCACATCA
Maob	GGCTGCTACACAACCTACTT	TCCAGTGTGAGGCTGTTTC
Idh2	GGAGAAGCCGGTAGTGGAGAT	GGTCTGGTCACGGTTTGGAA
Nkx6.1	AGAGAGCAGGCTTGGCCTATTC	GTCGTCAGAGTTCGGGTCCAG
Ngn3	TCTCAAGCATCTCGCCTCTTC-	ACAGCAAGGGTACCGATGAGA
Sox9	GTACCCGCATCTGCACAACG	GATTGCCCAGAGTGCTCGC
Actin	GTGACGTTGACATCCGTAAAGA	GTGACGTTGACATCCGTAAAGA
Primers for Rat		
MafB	ACCAAGGACGAGGTGATCC	CAGGTGATGTTTCTGCTGGA
Glucagon	GATCATTCCCAGCTTCCCAG	CTGGTAAAGGTCCCTTCAGC
Corin	TGCTTTGAAGGGAGAGAGGATG	ACCACACTGATGTCATAGTCTACC
Fam49a	CAATAAGTCGTAACCGCATCAAC	GCAGTCTGTGGTATTCTCTATGG
Cela1	AATGCCCAGCCTACGGAAG	CTCACGACCAGAGGACAGAC
Raptor	GATGGTGGTGGACTGGGAAC	TGAGTGAGCGGTGCGAATC
Slc2a2	ACACCAGCACATACGACACC	AGCCACCCACCAAAGAACG
Msln	CCCACTTCTTCTCCCTCATCTC	ACTCACTTGAAATCCATACACACC
Ppp1r1a	CGGAAGAAGATGACAAGGACTAC	TCTCAGTGGCTCCCTCAGG
Etv1	CTGTAACTCTTTCCCTCCTTTGC	GGTCGTGGTACTCCTGCTTG
Tspan12	GCTCTACGCCCTCAACCTG	GATGACCGCCTCTTCTACCC
Aass	CGCCATCATCATCTTGTCAGG	TTTGCTCCCAGTAGATTCCATTG
Pcsk1	GCTGGGCTATGACCTCTTGG	GCTGAGTCTCTTGGAACTGAAC
Cpe	GGCTACCTGGCAATCACAAAG	TCTTCCTTCTCCTCCTCCTTCC
Actin	AGGCCCCTCTGAACCCTAAG	GGAGCGCGTAACCCTCATAG
