# Supplementary material for: Raptor determines β-cell identity and plasticity independent of hyperglycemia in mice
Source: Nat Commun. 2020 May 21;11:2538. doi: 10.1038/s41467-020-15935-0 (PMC7242325; doi:10.1038/s41467-020-15935-0)
Supplement: Supplementary file 2 — Reporting Summary [file 41467_2020_15935_MOESM2_ESM.pdf]

nature research

Corresponding author(s): Qidi Wang, Guang Ning

Last updated by author(s): 2020-03-05

Reporting Summary

Nature Research wishes to improve the reproducibility of the work that we publish. This form provides structure for consistency and transparency in reporting. For further information on Nature Research policies, see Authors & Referees and the Editorial Policy Checklist.

Please do not complete any field with "not applicable" or n/a. Refer to the help text for what text to use if an item is not relevant to your study.
For final submission: please carefully check your responses for accuracy; you will not be able to make changes later.

Statistics

For all statistical analyses, confirm that the following items are present in the figure legend, table legend, main text, or Methods section.

n/a	Confirmed	
☐	☑	The exact sample size (*n*) for each experimental group/condition, given as a discrete number and unit of measurement
☐	☑	A statement on whether measurements were taken from distinct samples or whether the same sample was measured repeatedly
☐	☑	The statistical test(s) used AND whether they are one- or two-sided<br>*Only common tests should be described solely by name; describe more complex techniques in the Methods section.*
☐	☑	A description of all covariates tested
☐	☑	A description of any assumptions or corrections, such as tests of normality and adjustment for multiple comparisons
☐	☑	A full description of the statistical parameters including central tendency (e.g. means) or other basic estimates (e.g. regression coefficient) AND variation (e.g. standard deviation) or associated estimates of uncertainty (e.g. confidence intervals)
☑	☐	For null hypothesis testing, the test statistic (e.g. *F*, *t*, *r*) with confidence intervals, effect sizes, degrees of freedom and *P* value noted<br>*Give P values as exact values whenever suitable.*
☑	☐	For Bayesian analysis, information on the choice of priors and Markov chain Monte Carlo settings
☑	☐	For hierarchical and complex designs, identification of the appropriate level for tests and full reporting of outcomes
☑	☐	Estimates of effect sizes (e.g. Cohen's *d*, Pearson's *r*), indicating how they were calculated

Our web collection on statistics for biologists contains articles on many of the points above.

Software and code

Policy information about availability of computer code

Data collection	Microscope: ZEN(blue edition)/2.1, Immunoblot:Image Studio Software version 5.2 (LI-COR) ,qPCR analysis: QuantStudio 12K Flex Software/v1.2.2，mass: Meta-Morph version6.1 Immunofluorescent images：ImageJ software （ImageJ 1.50i）
Data analysis	Statistical analyses were performed using SPSS version 22.0.

For manuscripts utilizing custom algorithms or software that are central to the research but not yet described in published literature, software must be made available to editors/reviewers. We strongly encourage code deposition in a community repository (e.g. GitHub). See the Nature Research guidelines for submitting code & software for further information.

Data

Policy information about availability of data

All manuscripts must include a data availability statement. This statement should provide the following information, where applicable:

- Accession codes, unique identifiers, or web links for publicly available datasets
- A list of figures that have associated raw data
- A description of any restrictions on data availability

All data supporting the findings of this study are available with the article. Data from RNA-seq are available under GEO Series GSE130792. Microarray data has been deposited in the NCBI GEO repository with an accession ID GSE140224. Source data for Figs.1-6 and Supplementary Figs. 1-4, Supplementary Figs.6, 7 are provided with the paper as a Source Data File.

Field-specific reporting

Please select the one below that is the best fit for your research. If you are not sure, read the appropriate sections before making your selection.

☑ Life sciences ☐ Behavioural & social sciences ☐ Ecological, evolutionary & environmental sciences

Life sciences study design

All studies must disclose on these points even when the disclosure is negative.

Sample size	All sample sizes are indicated in the figures legends or in the Methods of our paper respectively. The sample size for each experiment was determined based on our previous experience, and were sufficient to support our conclusions with statistical significance.
Data exclusions	No data were excluded from analysis.
Replication	All results reported were successfully replicated. Exact numbers of biologically independent repetitions are given in our paper.
Randomization	All mice were randomly assigned to experimental groups (Animals used in experiments of this study were randomly grouped. IHC, IF and histology were performed and analyzed in a double blinding way). INS-1 cells were randomly assigned to different experimental groups.
Blinding	Investigators were blinded for most of the qualification experiments. During implantation of insulin pump, investigators were aware of the mice's genotyping.

Reporting for specific materials, systems and methods

Materials & experimental systems

n/a	Involved in the study
☐	☑ Antibodies
☐	☑ Eukaryotic cell lines
☑	☐ Palaeontology
☐	☑ Animals and other organisms
☑	☐ Human research participants
☑	☐ Clinical data

Methods

n/a	Involved in the study
☑	☐ ChIP-seq
☐	☑ Flow cytometry
☑	☐ MRI-based neuroimaging

Antibodies

Antibodies used

Antibodies used for Immunostaining(name; company catalog number; dilution) Guinea pig anti-Insulin,Dako A0564,1:800 Mouse anti-Glucagon ,Abcam K79bB10,1:800
Rabbit anti-Arx,A kind gift from Dr Kunio Kitamura,1:1000
Mouse anti-Nkx6.1,DSHB F55A10,1:1000
Rabbit anti-Pdx1,Abcam ab47267,1:1000
Rabbit anti-Somatostatin,Millipore MAB354,1:400
Rabbit anti-MafA,Bethyl laboratories IHC-00352,1:1000
Rabbit anti-Glut2,Millipore 400061,1:400
Rabbit anti-Pancreatic Polypeptide,Millipore AB939,1:400
Goat anti-GFP,Rockland-600-101-215M,1:400
Rabbit anti-insulin,Cell Signaling Technology 3014,1:500
Rabbit anti-Aldh1a3,Novus NBP2-15339,1:400
Rabbit anti-Ucn3,Sigma HPA038281,1:400
Rabbit anti-Ki67,Bethyl laboratories IHC-00375,1:500
Rabbit anti-MafB,Bethyl laboratories IHC-00351,1:100
Rabbit anti-proinsulin,A kind gift from Dr Min Liu ,1:200
Rabbit anti-CPE, GeneTex GTX33060, 1:1000

Antibodies used for Western Blotting(name; dilution; company;catalog number)
Rabbit anti-RAPTOR 1:1000, Cell Signaling Technology, #2280
Rabbit anti-PS6 (Ser240/244) 1:1000, Cell Signaling Technology, #5364
Rabbit anti-4E-BP1 1:1000, Cell Signaling Technology, #9644
Rabbit anti-TUBULIN 1:1000, Cell Signaling Technology, #2125
Guinea pig polyclonal anti-insulin antibody from Liuming Lab 1:1000

Secondary antibodies (name;dilution;catalog number;company)
Donkey anti-Mouse Alexa Fluor 594, 1:500, A-21203, Invitrogen
Donkey anti-Rabbit Alexa Fluor 594 ,1:500, A-21207, Invitrogen
Donkey anti-Rabbit Alexa Fluor 488 ,1:500, A-21206, Invitrogen
Goat anti-Guinea Pig Alexa Fluor 488, 1:500, A-11073, Invitrogen
Goat anti-Guinea Pig Alexa Fluor 647,1:500, A-21450, Invitrogen
Donkey anti-Goat Alexa Fluor 594, 1:500, A-11058, Invitrogen
Horseradish peroxidase-coupled goat-anti-rabbit IgG, 1:2000, #7074,Cell Signaling Technology

Validation

-Mouse anti-Glucagon ,Abcam K79bB10, the antibody is recommended for IF,IHC,RIA. Reactive against Mouse, Rat, Dog, Human, Pig, Common marmoset.https://www.abcam.cn/glucagon-antibody-k79bb10-ab10988.html
-Rabbit anti-Pdx1,Abcam ab47267, the antibody is recommended for IHC,IF,WB. Reactive against Mouse, Rat, Cow,Dog, Human. https://www.abcam.cn/pdx1-antibody-ab47267.html
-Rabbit anti-Somatostatin,Millipore MAB354, the antibody is recommended for IHC. Reactive against Mouse, Rat,Rabbit. https://www.merckmillipore.com/CN/zh/product/Anti-Somatostatin-Antibody-clone-YC7,MM_NF-MAB354
-Rabbit anti-MafA, Bethyl laboratories IHC-00352, the antibody is recommended for IHC. Reactive against Mouse. https://www.bethyl.com/product/IHC-00352?referrer=home&target=MafA
-Goat anti-GFP,Rockland 600-101-215M,the antibody is recommended for ELISA, WB, IHC. Reactive against Human, Mouse, Rat. https://rockland-inc.com/Product.aspx?id=46735
-Rabbit anti-insulin, Cell Signaling Technology 3014,the antibody is recommended for IF,IHC. Reactive against Human, Mouse, Rat. https://www.cst-c.com.cn/products/primary-antibodies/insulin-c27c9-rabbit-mab/3014
-Rabbit anti-Aldh1a3, Novus NBP2-15339,the antibody is recommended for IF,IHC. Reactive against Human, Mouse, Rat. https://www.novusbio.com/products/aldh1a3-antibody_nbp2-15339
-Rabbit anti-MafA, Bethyl laboratories IHC-00375,the antibody is recommended for IHC.Reactive against Mouse. https://www.biomol.com/products/antibodies/primary-antibodies/general/anti-ki-67-ihc-ihc-00375-t?number=IHC-00375
-Rabbit anti-CPE, GeneTex GTX33060,the antibody is recommended for WB,IF. Reactive against Human, Mouse. https://www.genetex.com/Product/Detail/Carboxypeptidase-E-antibody/GTX33060
-Rabbit anti-RAPTOR,Cell Signaling Technology 2280,the antibody is recommended for WB,IP. Reactive against Human, Mouse, Rat. https://www.cst-c.com.cn/products/primary-antibodies/raptor-24c12-rabbit-mab/2280
-Rabbit anti-PS6(Ser240/244),Cell Signaling Technology 5364, the antibody is recommended for WB,IF,IHC. Reactive against Human, Mouse, Rat. https://www.cst-c.com.cn/products/primary-antibodies/phospho-s6-ribosomal-protein-ser240-244-d68f8-xp-rabbit-mab/5364?site-search-type=Products
-Rabbit anti-4E-BP1,Cell Signaling Technology 9644,the antibody is recommended for WB,IF,IHC,IP. Reactive against Human, Mouse, Rat. https://www.cst-c.com.cn/products/primary-antibodies/4e-bp1-53h11-rabbit-mab/9644
-Rabbit anti-TUBULIN,Cell Signaling Technology 2125, the antibody is recommended for WB,IF,IHC. Reactive against Human, Mouse, Rat. https://www.cst-c.com.cn/products/primary-antibodies/a-tubulin-11h10-rabbit-mab/2125

Eukaryotic cell lines

Policy information about cell lines

Cell line source(s)	INS-1 cells were purchased from the CAMS Cell Culture Center (Beijing, China).
Authentication	INS-1 cells were validated by morphology and secretion of insulin.
Mycoplasma contamination	Cells have been routinely tested for mycoplasma and they were negative.
Commonly misidentified lines (See ICLAC register)	No commonly misidentified cell lines were used.

Animals and other organisms

Policy information about studies involving animals; ARRIVE guidelines recommended for reporting animal research

Laboratory animals	Raptorfl/fl and Rosa26-EGFP mice were purchased from The Jackson Laboratory. Raptorfl/fl, RIPCre, Rosa26-EGFP were then bred and maintained under specific pathogen free conditions. Matched male mice, aged 2-12 weeks old, were used.
Wild animals	No wild animals were used in this study.
Field-collected samples	This study did not involve collected samples from the field.
Ethics oversight	All animal experiments were approved by the Animal Care Committee of Shanghai Jiao Tong University School of Medicine.

Flow Cytometry

Plots

Confirm that:

☑ The axis labels state the marker and fluorochrome used (e.g. CD4-FITC).

☑ The axis scales are clearly visible. Include numbers along axes only for bottom left plot of group (a 'group' is an analysis of identical markers).

☑ All plots are contour plots with outliers or pseudocolor plots.

☑ A numerical value for number of cells or percentage (with statistics) is provided.

Methodology

Sample preparation	Single-cell suspensions from islets were generated by macerating the tissues through a 70mm nylon mesh.
Instrument	Beckman-Coulter MoFlo XDP flow cytometer for cell sorting.
Software	We analyzed the acquired samples with Summit using FlowJo 10 (TreeStar).
Cell population abundance	To purify β cells, single-cell suspensions from islets were sorted using a MoFlo XDP flow cytometer (Beckman-Coulter) with purities of 95%.
Gating strategy	FSC/SSC gates were used to selected live cells.

☑ Tick this box to confirm that a figure exemplifying the gating strategy is provided in the Supplementary Information.
